# WRA-Net: Wide Receptive Field Attention Network for Motion Deblurring in Crop and Weed Image

**DOI:** 10.34133/plantphenomics.0031

**Published:** 2023-04-05

**Authors:** Chaeyeong Yun, Yu Hwan Kim, Sung Jae Lee, Su Jin Im, Kang Ryoung Park

**Affiliations:** Division of Electronics and Electrical Engineering, Dongguk University, 30 Pildong-ro 1-gil, Jung-gu, Seoul 04620, Republic of Korea.

## Abstract

Automatically segmenting crops and weeds in the image input from cameras accurately is essential in various agricultural technology fields, such as herbicide spraying by farming robots based on crop and weed segmentation information. However, crop and weed images taken with a camera have motion blur due to various causes (e.g., vibration or shaking of a camera on farming robots, shaking of crops and weeds), which reduces the accuracy of crop and weed segmentation. Therefore, robust crop and weed segmentation for motion-blurred images is essential. However, previous crop and weed segmentation studies were performed without considering motion-blurred images. To solve this problem, this study proposed a new motion-blur image restoration method based on a wide receptive field attention network (WRA-Net), based on which we investigated improving crop and weed segmentation accuracy in motion-blurred images. WRA-Net comprises a main block called a lite wide receptive field attention residual block, which comprises modified depthwise separable convolutional blocks, an attention gate, and a learnable skip connection. We conducted experiments using the proposed method with 3 open databases: BoniRob, crop/weed field image, and rice seedling and weed datasets. According to the results, the crop and weed segmentation accuracy based on mean intersection over union was 0.7444, 0.7741, and 0.7149, respectively, demonstrating that this method outperformed the state-of-the-art methods.

## Introduction

Increasing crop productivity is important because food security is an important issue worldwide. However, it meets challenges such as insufficient manpower, abnormal climate, and water shortage. Precision agriculture using plant phenotyping is one of the ways to overcome these problems and improves crop productivity by increasing harvest efficiency [1]. Semantic segmentation is the computer vision task used in image-based phenotyping. Previous researches [2–4] studied semantic segmentation-based plant phenotyping, and crop and weed semantic segmentation was also performed. This is very important especially in techniques for increasing the efficiency of spraying herbicides. Traditional methods of controlling weeds have problems such as polluting crops or wasting herbicides, which negatively impact the environment due to poor accuracy. Moreover, as these techniques are inefficient, manpower is insufficient and cannot keep up with the growing crop demand. Farming robots that target weeds to spray herbicides can solve these problems. In this form of smart farming, the main problem to be solved by image-based phenotyping is accurately recognizing crops and weeds [5]. In other words, weeds must be accurately separated from crops to spray herbicides on weeds without harming the crops. Crop and weed semantic segmentation accurately detects the regions of an object in pixels, making it suitable for crop and weed localization. However, when capturing crop and weed images with a camera before segmentation, motion blur occurs due to various causes (e.g., vibration or shaking of farming robots, shaking of crops and weeds). Motion blur severely degrades the quality of the captured crop and weed images, reducing the accuracy of high-level vision tasks (e.g., object detection, recognition, and segmentation) [6]. Figure S1 visually compares the results of semantic segmentation by U-Net [7] for a blurred and sharp image. Unlike the sharp image, crop and weed regions were not accurately recognized in the blurred image. Accordingly, a restoration process for motion-blurred images is necessary to segment crops and weeds more accurately. However, previous crop and weed segmentation studies were performed without considering motion blur. This study proposes a method that restores the motion-blurred images to perform crop and weed segmentation, making this the first study on crop and weed segmentation considering motion blur.

This study classified prior research related to crop and weed segmentation not considering motion blur and crop and weed segmentation considering motion blur.

### Crop and weed segmentation not considering motion blur

This subsection classifies the research on crop and weed segmentation not considering motion blur into (a) research based on handcrafted features such as color, shape, and texture, and (b) research based on deep features extracted by a deep learning model and describes the two.

#### Handcrafted feature-based methods

In a study on crop and weed discrimination in celery cabbage and broccoli fields [8], the researchers converted images in red, green, and blue (RGB) color space to the hue, saturation, and intensity (HSI) color space, which is similar to how the eye perceives images. They used the H and S channel values normalized to a [0, 1] range as features for crop and weed discrimination through Mahalanobis distance-based classification [9], which has the advantage of scale invariance. These features were not directly related to changes in various light environments, thus enabling robust crop and weed segmentation in various light environments. In a study on a detection system for sugar beet plants and weeds [10], the researchers used RGB images and near-infrared (NIR) images as input. Using the high reflectivity of chlorophyll in the NIR images, they generated normalized difference vegetation index (NDVI) images [11] with the R channel and NIR channel. They then created a masked image by separating the crop and weed from the background through threshold-based classification in the NDVI image. In addition, they used key point-based feature extraction and object-based feature extraction in the masked region to extract statistical features and shape features and classify the crops and weeds through random forest classification. Another study investigated maize crop and weed discrimination [12], which applied the excess green [13] method to separate maize crops and weeds from the soil region and then generated binary images with the Otsu threshold. They applied erosion and dilation techniques to remove noise and blur from the binary image, i.e., create a mask for the crop and weed positions. The researchers extracted 12 color indices from the region of the color image masked by this mask as features to classify the maize and weeds through support vector data description, a one-class classifier [14]. These methods have fast inference time and low computing power requirements but suffer from relatively low segmentation accuracy.

#### Deep feature-based methods

Milioto et al. [15] proposed a deep leaning-based crop and weed segmentation model modifying SegNet [16] and Enet [17]. The researchers replaced the convolutional layers of SegNet with residual blocks designed to reduce the computational complexity, enabling effective model training and decreasing the inference time. In addition, they used images that concatenated 14 channels obtained by transforming the RGB images as input images, which allows features unaffected by the capture environment to be used as input, thereby improving the crop and weed segmentation accuracy in various environments. Another study [5] configured the encoder modifying a fully convolutional DenseNet [18], and used RGB images or both RGB and NIR images as input. This study also proposed a decoder that plays a role in stem detection and used it to detect stems by sharing the encoder’s output. A previous study [19] presented a multistage method that separately detects weeds and crops and proposed a new convolutional neural network model called CED-Net (cascaded encoder–decoder network). They constructed a 4-stage backbone model modifying U-Net [7] and performed weed segmentation by concatenating the 1-stage and 2-stage outputs in the channel direction. Crop segmentation was then performed through the same method in the 3-stage and 4-stage, thus improving segmentation accuracy with a crop and weed detection technique configured in stages.

A study using various types of images captured by unmanned aerial vehicles as input performed crop and weed segmentation using the red channel of RGB, NIR, and NDVI images as input [20]. They configured the encoder of this model with VGG-16 [21], with a U-Net-based architecture for the decoder. The crop and weed segmentation accuracy was enhanced by concatenating images of various formats in the channel direction and using them as input. A study that proposed patch-unit crop and weed segmentation for input images [22] used a modified U-Net. Patches of crop and weed images were used as model input to consider the local region and observe the effect of data augmentation. Through this process, they extracted detailed features on the shapes of the crops and weeds, thus improving the segmentation accuracy. A study using RGB and NIR images as input [23] applied a structure connecting a universal function approximation block (UFAB) comprising several convolutional blocks and a residual net (ResNet)-50 [24] as the encoder. Three feature maps are passed from the encoder to the decoder, and after applying a bridge attention block (BAB), 2 feature maps are passed to the decoder [25,26]. The decoder sequentially comprises a BAB, deconvolution [27], and spatial pyramid refinement block and performs segmentation based on the output features of the encoder. In addition, 3 auxiliary losses are configured with the BAB outputs and features of the last layer, enabling the outputs of the last layer and the BAB outputs to be applied to learning. Finally, a previous study [28] proposed a 2-stage model using 2 U-Net models called the multitask semantic segmentation–convolutional neural network. In this method, crops and weeds are considered as one object in the first stage, the object region and background region are separated, and attention is applied to the input image using this. The attention-assigned input becomes the input of the second stage. In the second stage, crop and weed as well as object regions are segmented. The loss for segmenting crop and weed and that for segmenting object are separated to prevent background-biased learning, thus improving segmentation performance. While these studies yield higher segmentation accuracy than handcrafted feature-based methods, they do not consider motion blur, which frequently occurs in camera images during crop and weed segmentation.

### Crop and weed segmentation considering motion blur

As examined above, no previous research on crop and weed segmentation considered motion blur, making this the first study on crop and weed segmentation considering motion blur. Motion blur occurs from various causes while capturing images; hence, it must be considered to improve segmentation accuracy when applied to actual agriculture sites. This study uses a wide receptive field attention network (WRA-Net) to restore crop and weed images with motion blur and then performs segmentation with U-Net. WRA-Net is a deep-learning model for motion-blurred image restoration designed for crop and weed images. Table S1 summarizes the advantages and disadvantages of existing methods and the proposed method. The contributions of this study are as follows.

• As the first study on crop and weed segmentation considering motion blur, we propose a method that performs segmentation after restoring the motion-blurred image. Hence, we propose WRA-Net, which restores motion-blurred images.
• WRA-Net includes a lite wide receptive field attention residual block (Lite WRARB) proposed here and an encoder part comprising convolutional block (Conv Block) 1 and 2. Lite WRARB computes modified depthwise separable convolutional block (mDSCB) groups in parallel and then concatenates them. Through this, the features of various receptive fields can be extracted.
• The decoder part of WRA-Net comprises an architecture that combines an upsample and feature aggregation part containing pixel shuffle with a deformable residual block (Deformable ResBlock) containing deformable convolution. This architecture enables it to perform convolutional operations at flexible sampling points, thereby improving restoration performance.
• The motion-blurred images generated here, developed model, and code are made available on GitHub for other researchers to perform fair performance evaluations [29].

The structure of this paper is as follows. Materials and Methods describes the proposed method, and Results includes experimental results. Finally, Discussion contains a summary of this paper and future research directions as a conclusion.

## Materials and Methods

### Proposed method

**Fig. 1.**
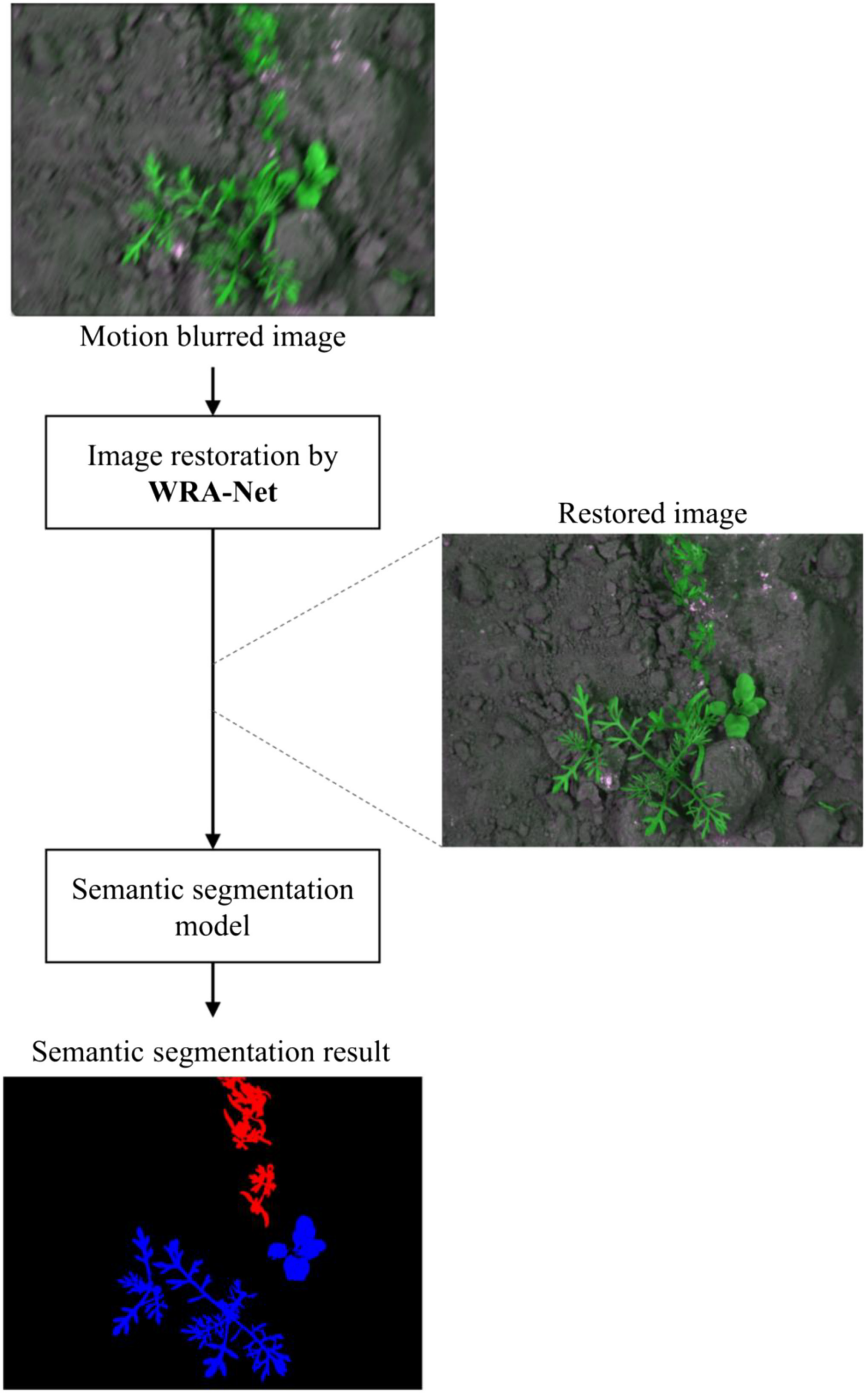
Flowchart of the overall system.

This subsection describes a system that restores motion-blurred images and performs crop and weed segmentation through the WRA-Net proposed here. Figure 1 shows a flowchart of this system.

**Fig. 2.**
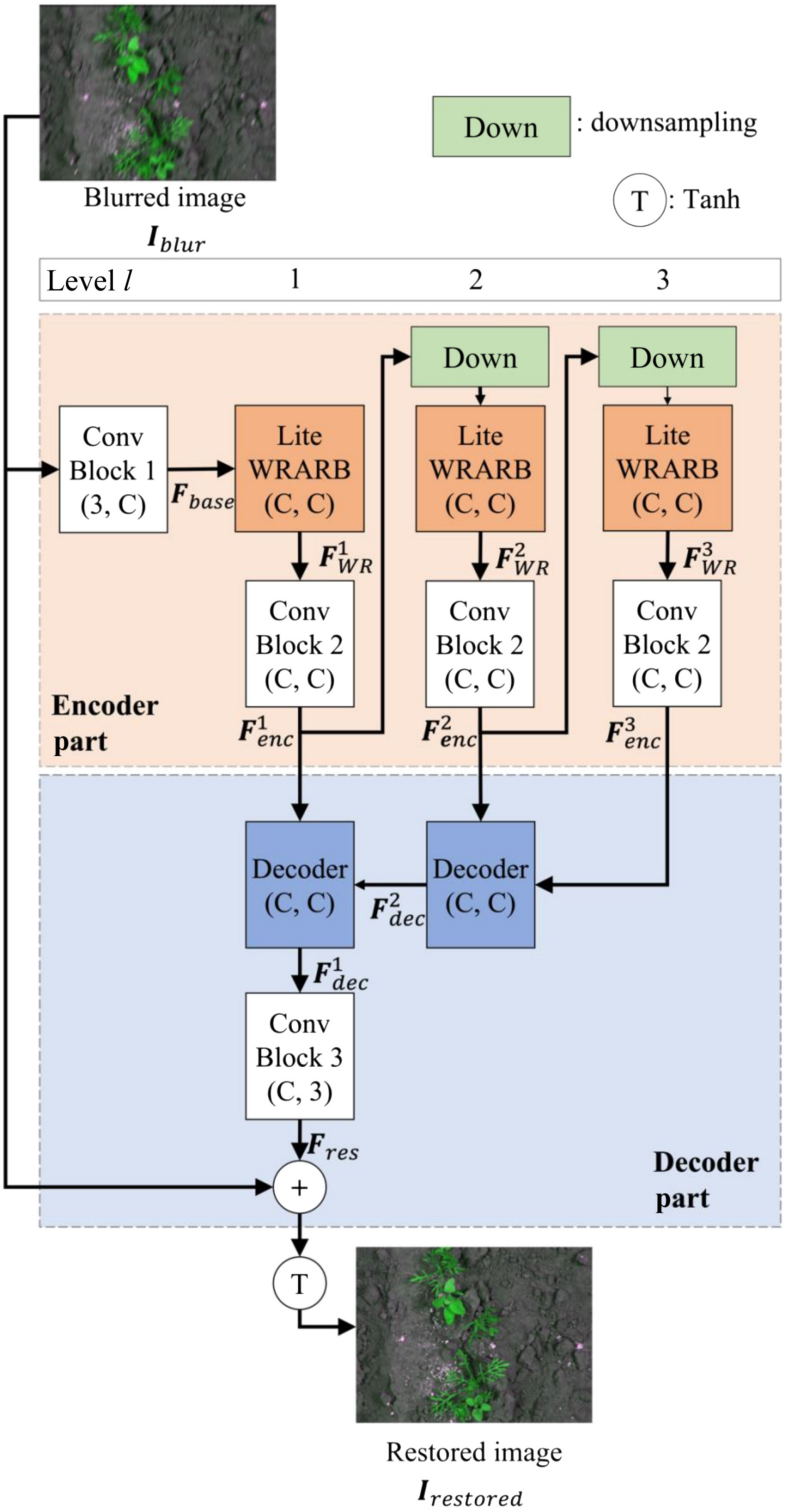
Overall structure of the proposed WRA-Net; the number of channels of input and the number of channels of the output of each module are expressed as (3, C), (C, C), or (C, 3).

Figure 2 shows the overall structure of the proposed WRA-Net. WRA-Net extracts deep features from the blurred image ***I****_blur_* in the encoder part and passes them to the decoder part. The decoder part generates restored images based on deep features. The pixel values of the input and output images of WRA-Net have a range of [−1, 1]. The main module of the encoder part is Lite WRARB, which extracts deep features with various receptive fields and then emphasizes important features with an attention gate. The main module of the decoder part is the decoder module, which comprises an upsample and feature aggregation part and Deformable ResBlock. The upsample and feature aggregation part doubles the height and weight of the output features of the previous decoder block through pixelshuffle, receives the output of the encoder, and extracts the aggregated features, in which the 2 features are aggregated. Thereafter, Deformable ResBlock samples the aggregated features in a flexible receptive field through deformable convolution operations, thus improving the quality of the restored image. Finally, learning is focused on the residue through a skip connection from the input to the output. The detailed architecture of WRA-Net is divided into encoder and decoder parts, which is explained in the next subsections.

#### Encoder part

In the encoder part of Fig. 2, feature extraction and downsampling are repeated through Conv Block 2 and Lite WRARB. The output features of Conv Block 2 in the encoder part are passed to the decoder part. Figures S2 and S3 present the detailed structure of the modules forming the encoder part. The downsampling layer in Fig. 2 is a convolutional layer with a filter size of 2 × 2 and a stride of 2. All activation functions of the encoder part are rectified linear units (ReLU). In addition, all normalization layers of the encoder part adopt instance normalization [30]. The reason for this is that most research on image restoration tasks, including the experiments here, use small image patches and apply a small batch size when training the network [31], which makes the statistics of batch normalization unstable [32–34].

##### Conv Block 1

Conv Block 1 in Fig. S3A passes the blurred image ***I***_***blur***_ ∈ *ℝ*^*H* × *W* × 3^ with 3 channels through 2 convolutional layers and then extracts the deep features ***F***_***base***_ ∈ *ℝ*^*H* × *W* × *C*^ with *C* channels. *H* and *W* indicate the height and width of the input image, respectively. ***F***_***base***_ is passed to the first Lite WRARB. While all experiments here were performed with *C* = 128, this hyperparameter can be adjusted to other values depending on the task.

##### Lite WRARB

Lite WRARB comprises a feature extraction part consisting of mDSCB, attention gate, and learnable skip connection. Figure S2 shows a detailed architecture of Lite WRARB.

###### mDSCB

Figure S3B shows the detailed architecture of mDSCB. mDSCB is a convolutional block comprising depthwise separable convolution [35]. Depthwise separable convolution separates the standard convolutional layer with weights ***w* ∈** *ℝ*^*k* × *k* × *i* × *o*^ into a standard convolutional layer with a kernel size of 1 and a depthwise convolutional layer with a kernel size of *k* when the kernel size is *k* × *k*, the number of channels of input features is *i*, and the number of channels of output features is *o*. The weight of the standard convolutional layer with a kernel size of 1 is ***w***_1_ ∈ *ℝ*^1 × 1 × *i* × *o*^, and the weight of the depthwise convolutional layer is ***w****_d_* ∈ *ℝ*^*k* × *k* × 1 × *o*^; hence, it has much fewer parameters than the standard convolutional layer. mDSCB applies *k* = 3 separable depthwise convolution to the input features and then applies instance normalization and ReLU function to generate output features.

###### Attention gate

Figure S3C shows the detailed architecture of the attention gate. The attention gate receives output ***F****^l^_fe_* ∈ *ℝ*^*H_in_* × *W_in_* × *C*^ of the feature extraction part as input and generates pointwise attention weights ***W****_attention_* ∈ *ℝ*^*H_in_* × *W_in_* × *C*^. *l* indicates the level of the encoder. The attention gate computes ***W****_attention_* including channel-specific importance values per point of ***F***^***l***^*_fe_* through pointwise convolution. As this process is trainable, it is optimized for the dataset during training. *H_in_* and *W_in_* are the height and width of the input feature maps $I_{\mathrm{WR}}^{l}$ of Lite WRARB, respectively. Multiplying ***F***^***l***^*_fe_* by ***W****_attention_* produces $F_{\mathrm{attention}}^{l}$, in which the important features of ***F***^***l***^*_fe_* are emphasized. This operation is represented by Eq. 1. “∘” in Eq. 1 refers to the Hadamard product.

$$\begin{array}{c} \sigma (\mathrm{Conv}2D(\mathrm{ReLU}(\mathrm{Conv}2D(F_{\mathrm{fe}}^{l},w_{1}))),w_{2})\circ \\ F_{\mathrm{fe}}^{l}=W_{\mathrm{attention}}\circ F_{\mathrm{fe}}^{l}=F_{\mathrm{attention}}^{l} \end{array}$$ (1)

###### Learnable skip connection

As Lite WRARB is a residual architecture [24], the input feature maps $I_{\mathrm{WR}}^{l}$ added by $F_{\mathrm{attention}}^{l}$ become the output feature maps $F_{\mathrm{WR}}^{l}$ of Lite WRARB. The residual architecture mitigates the gradient vanishing/exploding problem through skip connection to stabilize training and prevent information loss [24,36]. Accordingly, Lite WRARB, the main module of the encoder part, was configured as a residual architecture to stabilize training and pass information from the initial layer to deeper layers. However, in terms of passing information, it may be inefficient to pass the features without modification. As the input of WRA-Net is a motion-blurred image, although it is important to pass the overall color and spatial information, the motion blur characteristics, which reduce the semantic segmentation accuracy, should not be passed. Accordingly, as shown in Fig. S2, learnable parameters **α ∈** *ℝ*^**1 × 1 × C**^ were added to the skip connection to adjust the degree of feature transmission. The values of channel *i* of **α** are multiplied by channel *i* of $I_{\mathrm{WR}}^{l}$. Considering the actual operation is performed by copying the values of **α** according to the spatial dimension of $I_{\mathrm{WR}}^{l}$, the operation of learnable skip connection can be expressed as the Hadamard product of **α** and $I_{\mathrm{WR}}^{l}$, as shown in Eq. 2. Values of **α** are initialized to zero before training. “∘” in Eq. 2 refers to the Hadamard product.

$$F_{\mathrm{attention}}^{l}+\alpha \circ I_{\mathrm{WR}}^{l}=F_{\mathrm{WR}}^{l}$$ (2)

Thus far, we have explained the submodules that constitute Lite WRARB. The flow of Lite WRARB is as follows. Lite WRARB inputs the input feature maps into 4 mDSCB groups in the feature extraction part and then concatenates the resulting output feature maps in the channel direction. The mDSCB groups comprise 1, 2, 3, and 4 mDSCB modules. Each group is defined as mDSCB group *n*, where *n* is the number of mDSCBs in the group. The output features of each mDSCB group have a receptive field of (1 + 2*n*). Accordingly, in the feature extraction part of Lite WRARB, feature maps with various receptive fields, the outputs of the mDSCB groups, are combined to generate concatenated feature maps ∈*ℝ*^*H_in_* × *W_in_* × 4*C*^ with wide receptive fields. Note that these feature maps are meaningful because they have information on various receptive fields beyond a wide receptive field. These concatenated feature maps become $F_{\mathrm{fe}}^{l}\in {\mathbb{R}}^{H_{\mathrm{in}}\times W_{\mathrm{in}}\times C}$ through pointwise convolution. If the input of Lite WRARB and the operation of mDSCB group *n* are defined as ***x*** and *f_n_*(***x***), respectively, the feature extraction part can be defined as in Eq. 3.

$$\mathrm{ReLU}\left(\mathrm{InstanceNorm}\left(\mathrm{Conv}2D(\mathrm{Concat}(f_{1}\left(x\right),f_{2}\left(x\right),f_{3}\left(x\right),f_{4}\left(x\right)),w)\right)\right)=F_{\mathrm{fe}}^{l}$$ (3)

As shown in Eq. 1, the attention weights ***W****_attention_* generated by attention gate are multiplied by $F_{\mathrm{fe}}^{l}$ to become features $F_{\mathrm{attention}}^{l}$, which emphasize important values. Then, $F_{\mathrm{WR}}^{l}$ applying a learnable skip connection becomes the output of Lite WRARB, as shown in Eq. 2.

##### Conv Block 2

Conv Block 2 in Fig. S3D is a convolutional block that sequentially performs the convolutional layer, instance normalization, and ReLU function. Conv Block 2 is located after Lite WRARB in the encoder part and refines the output features $F_{\mathrm{WR}}^{l}$ of Lite WRARB to generate $F_{\mathrm{enc}}^{l}$.

#### Decoder part

This subsection describes the decoder part in detail. The decoder part in Fig. 2 comprises a decoder module and Conv Block 3. Figure S4 shows the structure of the decoder module in the decoder part, and Fig. S5 shows the structure of Conv Block 3. Batch normalization was applied for the normalization layers of the decoder part regarding the ablation studies in the Ablation studies subsection under the Testing with the CWFID dataset section (Table 4) and experimental results of Ref. [31].

##### Decoder module

As shown in Fig. S4, the decoder module is broadly divided into 2 parts: upsample and feature aggregation part and Deformable ResBlock.

##### Upsample and feature aggregation part

The upsample and feature aggregation part of the *l* level decoder module receives the output of the previous layer and output $F_{\mathrm{enc}}^{l}$ of Conv Block 2 in level *l* of the encoder part as input. The output of the previous layer is upsampled through pixelshuffle [37], and the upsampled features and $F_{\mathrm{enc}}^{l}$ are concatenated in the channel direction. Then, 3 × 3 convolution is performed on the concatenated features to extract $F_{\mathrm{agg}}^{l}$, which is the aggregated features of the 2 inputs.

##### Deformable ResBlock

The output $F_{\mathrm{agg}}^{l}$ of the upsample and feature aggregation part is passed to the Deformable ResBlock as input. Deformable ResBlock comprises a deformable convolutional layer, ReLU, and a convolutional layer with 3 × 3 filters. The deformable convolutional layer, proposed for the object detection task, can flexibly handle geometric transformations in objects by applying learnable offsets and modulation to the convolutional filter [38]. Here, we used this layer to restore pixels that shifted from their original position due to motion blur. Specifically, this layer is applied to the decoder module and the features extracted from the encoder part are calculated with flexible sampling points, thus potentially improving the quality of the restored image. Deformable ResBlock takes $F_{\mathrm{agg}}^{l}$ as input and applies deformable convolution, ReLU function, and 3 × 3 convolution, then add $F_{\mathrm{agg}}^{l}$ through the residual connection. Equation 4 is an equation of Deformable ResBlock.

$$\begin{array}{c} \mathrm{Conv}2D(\mathrm{ReLU}(\mathrm{DeformableConv}2D(F_{\mathrm{agg}}^{l},w_{\mathrm{deform}})),w_{\mathrm{conv}}))+\\ F_{\mathrm{agg}}^{l}=F_{\mathrm{dec}}^{l} \end{array}$$ (4)

##### ConvBlock 3

As shown in Fig. S5, convolutional block 3 (ConvBlock 3) serves to refine the output features $F_{\mathrm{dec}}^{1}$ of the last decoder module in the decoder part and reduce the number of channels to match the number of channels of the input image. It comprises 3 × 3 convolutional layers, and each layer sequentially reduces the number of channels of $F_{\mathrm{dec}}^{1}\in {\mathbb{R}}^{H\times W\times C}$ to C//2, C//4, and 3. C//n means the quotient of C divided by n. The output of ConvBlock 3 is ***F***_res_ ∈ *ℝ*^*H* × *W* × 3^. Restored image ***I****_restored_*, the final output of WRANet, is the result of adding ***F***_res_ and input image ***I****_blur_* through the residual connection and then applying the tanh function.

$$\tanh\left(F_{\mathrm{res}}+I_{\mathrm{blur}}\right)=I_{\mathrm{restored}}$$ (5)

#### Loss function

*SSIM* loss [39] and L1 loss were combined and used as the loss function for the training of WRA-Net, expressed in Eq. 7. ***I****_restored_* ∈ *ℝ*^*H* × *W* × 3^ and ***I****_target_* ∈ *ℝ*^*H* × *W* × 3^ refer to the image restored with WRA-Net and the sharp original image without motion blur, respectively, and *H* and *W* indicate the height and width of the images. The metric *SSIM* [40] is calculated as in Eq. 6; values closer to 1 indicate good restoration; hence, it is used as (1 − *SSIM*) in the loss equation. In Eq. 6, *μ_x_*, *μ_y_*, *σ_x_*, *σ_y_*, and *σ_xy_* indicate the mean of *x*, mean of *y*, the standard deviation of *x*, standard deviation of *y*, and covariance of *x* and *y*, respectively. *m* and *n* are constants to prevent instability when the denominator of the formula approaches 0. Here, *m* = 2.55 and *n* = 7.5 were applied with reference to previous research results [40]. L1 loss is the L1 distance of ***I****_restored_* and ***I****_target_*. *λ*_1_ is a hyperparameter for adjusting the ratio of the 2 losses; in this study, the optimal *λ*_1_ to obtain the highest semantic segmentation accuracy using training data was determined as 0.84.

$$\mathrm{SSIM}(x,y)=\frac{\left(2\mu_{x}\mu_{y}+m^{2}\right)\left(2\sigma_{\mathrm{xy}}+n^{2}\right)}{\left(\mu_{x}^{2}+\mu_{y}^{2}+m^{2}\right)\left(\sigma_{x}^{2}+\sigma_{y}^{2}+n^{2}\right)}$$ (6)

$$L_{R}=\lambda_{1}\left(1-\mathrm{SSIM}(I_{\mathrm{restored}},I_{\mathrm{target}})\right)+\left(1-\lambda_{1}\right){\left|I_{\mathrm{restored}}-I_{\mathrm{target}}\right|}_{1}$$ (7)

Dice loss [41] was used to train the segmentation model as expressed in Eq. 8. *Cls* is the number of classes; 3 (background, crop, and weed) were used in all experiments of this study. ***P*** ∈ *ℝ*^*H* × *W* × *Cls*^ and ***L*** ∈ *ℝ*^*H* × *W* × *Cls*^ signify the semantic segmentation result and ground truth label map, respectively, and ***P****_c_* ∈ *ℝ*^*H* × *W*^ and ***L****_c_* ∈ *ℝ*^*H* × *W*^ are the values corresponding to class *c*.

$$L_{\mathrm{dice}}=1-\frac{1}{\mathrm{Cls}}\sum_{c=1}^{\mathrm{Cls}}\frac{2\times \sum_{i=1}^{H\times W}P_{c}\left(i\right)\times L_{c}\left(i\right)}{\sum_{i=1}^{H\times W}\left(P_{c}\left(i\right)+L_{c}\left(i\right)\right)}$$ (8)

#### Patch-based restoration

When restoring blurred images, using the entire image as input has 2 problems. First, if inputting a part of the image as patch cut during training and the entire image during testing, the statistics of the image patches and the entire image becomes different, leading to poor restoration performance [42]. Second, extensive graphics processing unit (GPU) memory is required to restore images with a high resolution like the datasets used here. As such, when testing the proposed method, we applied a method that divides the input image into patches and then restores it.

### Experimental dataset and setup

The experiment was conducted with 3 public datasets: the crop/weed field image dataset (CWFID) dataset [43], the BoniRob dataset [44], and the rice seedling and weed dataset [45]. These datasets are for segmentation of crops and weeds; they comprise a crop and weed image and its corresponding pixel-wise ground truth label pair. The CWFID dataset contains labels for carrot and weed, and the images were captured by a JAI AD-130GE camera [46] with a resolution of 1,296 ×966 pixels. The BoniRob dataset contains data captured using a farming robot, with classes for sugar beet plants, dicot weeds, and grass weeds. In this study, we used images with a resolution of 1,296 × 966 pixels and labels for sugar beet plants and grass weeds. Finally, the rice seedling and weed dataset was collected with an IXUS 1000 HS (Lens model of EF-S 36–360 mm f/3.4–5.6) camera [47], and the images have a resolution of 912× 1,024 pixels. There are labels for rice and *Sagittaria trifolia* weed in this dataset. Figure S6 shows examples of these datasets.

Table S2 shows the number of images used for training, validation, and testing in these 3 public datasets. To train WRA-Net, the above dataset images were randomly cropped into patches with a resolution of 256 × 256 pixels. The experiments were performed on a desktop computer using Ubuntu20.04 with an Intel® Core™ i7-7700 central processing unit (CPU), 15 GB of RAM, NVIDIA GeForce GTX 1070 TI [48], and NVIDIA GeForce RTX 3060 [49]. The algorithms of the proposed method were implemented in Pytorch 1.12 [50].

### Motion-blurred datasets for crop and weed image restoration

A blurred dataset is required to perform motion blur restoration of crop and weed images. The CWFID, BoniRob, and rice seedling and weed datasets used here do not contain motion-blurred data, and there are no existing motion-blurred crop and weed datasets. Accordingly, to construct a dataset for motion-blurred crop and weed restoration, we generated random and nonlinear motion blur through the method proposed by previous research [6] to build a motion-blurred crop and weed database.

### Evaluation metrics

In this experiment, the semantic segmentation performance was measured through *IOU* of each class (background, weed, and crop), *mIOU, Recall*, *Precision*, and *F*1 *score*. The restoration performance for motion-blurred images was measured through a structural similarity index map (*SSIM*) and peak signal-to-noise ratio (*PSNR*), which quantitatively evaluates the similarity with the original image. *PSNR* is shown in Eq. 10, and *SSIM* is shown in Eq. 6. In Eqs. 9 and 10, ***I****_restored_* is the restored image, and ***I****_target_* is the original image without motion blur. Equations 11 to 15 show the evaluation metrics for semantic segmentation accuracy. *Cls* indicates the number of classes, which is *Cls* = 3 here. True positive (*TP*) and true negative (*TN*) refer to cases where the true label and false label match the prediction, respectively. False positive (*FP*) and false negative (*FN*) refer to cases where a false label is incorrectly predicted as true and a true label is incorrectly predicted as false, respectively. *H* and *W* indicate the height and width of the image. *IOU_i_* in Eq. 12 is the IOU of class *i*. *Recall* and *Precision* were calculated for each class and then evaluated through the average values. The values were also used to calculate *F*1 *score*.

$$\mathrm{MSE}=\frac{\sum_{i=1}^{W}\sum_{j=1}^{H}{\left(I_{\mathrm{restored}}(i,j)-I_{\mathrm{target}}(i,j)\right)}^{2}}{H\times W}$$ (9)

$$\mathrm{PSNR}=10\log_{10}\left(\frac{\mathrm{Max}{\left(I_{\mathrm{restored}}\right)}^{2}}{\mathrm{MSE}}\right)$$ (10)

$$\mathrm{IOU}=\frac{\mathrm{TP}}{\mathrm{TP}+\mathrm{FN}+\mathrm{FP}}$$ (11)

$$\mathrm{mIOU}=\frac{\sum_{i=1}^{\mathrm{Cls}}\mathrm{IO}U_{i}}{\mathrm{Cls}}$$ (12)

$$\mathrm{Recall}=\frac{\mathrm{TP}}{\mathrm{TP}+\mathrm{FN}}$$ (13)

$$\mathrm{Precision}=\frac{\mathrm{TP}}{\mathrm{TP}+\mathrm{FP}},$$ (14)

$$F1\;\mathrm{score}=\frac{2\times \mathrm{Precision}\times \mathrm{Recall}}{\mathrm{Precision}+\mathrm{Recall}}$$ (15)

## Results

### Training details

The proposed WRA-Net was trained through Adam optimizer [51] with an initial learning rate of 1 × 10^−4^, and the learning rate is steadily decreased to 1 × 10^−7^ using the cosine annealing strategy [52]. Table S3 explains the hyperparameters used in training. β_1_, β_2_ are hyperparameters for Adam optimizer. We trained WRA-Net on 256 × 256 randomly cropped patches with a batch size of 2 for 450 epochs.

Figure S7 shows a graph of changes in train and validation losses according to the epoch in the training process of WRA-Net. As the epoch increased, the loss for the train set converged to a sufficiently small value, indicating that WRA-Net was sufficiently trained on the training data. Moreover, the loss for the validation set also converged to a small value as the epoch increased, indicating that WRA-Net was not overfitted on the training data.

The training scheme used here comprises 2 steps. The first step is to train the proposed WRA-Net, a blurred image restoration model. We then freeze the parameters of WRA-Net and, in the second step, train the semantic segmentation model with the restored images. U-Net [7] was selected for the semantic segmentation model according to the experiment’s results in the Ablation studies subsection under the Testing with the CWFID dataset section (Table S5). Table S4 presents the training hyperparameters of U-Net. Adam optimizer with a learning rate of 1 × 10^−5^ was used to optimize U-Net.

### Testing of proposed method

#### Testing with the CWFID dataset

##### Experiments with various semantic segmentation models

This subsection compares the performance of the semantic segmentation models before and after motion blur restoration with WRA-Net, shown in Table S5. All models showed substantially improved performance after restoration compared to before restoration, indicating that WRA-Net can restore the detailed features of crop and weed images needed for semantic segmentation that was lost due to motion blur. U-Net yielded the highest performance of the 4 semantic segmentation models; hence, it was used as the semantic segmentation model in all later experiments.

##### Ablation studies

This paper conducted ablation studies from 3 perspectives. The first is an experiment on the main idea of the proposed WRA-Net that modifies or removes Lite WRARB and Deformable ResBlock and compares the resulting performance. The cases of this experiment are specifically described as follows.

**Case 1**: Excludes **α**, the learnable skip connection parameter of Lite WRARB in Fig. S2.

**Case 2**: Excludes the application of attention weights through the attention gate of Lite WRARB in Fig. S2.

**Case 3**: Replaces the deformable convolutional layer of Deformable ResBlock with the standard convolutional layer in the decoder module in Fig. S4.

**Case 4**: Sets the number of mDSCB groups of Lite WRARB in Fig. S2 to 2, and the number of mDSCB modules of mDSCB groups to 1 and 2, respectively.

**Case 5**: Sets the number of mDSCB groups of Lite WRARB in Fig. S2 to 2, and the number of mDSCB modules of mDSCB groups to 3 and 4, respectively.

**Case 6**: Sets the number of mDSCB groups of Lite WRARB in Fig. S2 to 3, and the number of mDSCB modules of mDSCB groups to 1, 2, and 3, respectively.

**Case 7**: Sets the number of mDSCB groups of Lite WRARB in Fig. S2 to 3, and the number of mDSCB modules of mDSCB groups to 2, 3, and 4, respectively.

**Case 8**: Proposed WRA-Net.

This is described briefly in Table S6.

Tables 1 and 2 present the results of this experiment. The performance for image quality and semantic segmentation is shown. Among the 7 ablation cases, Case 3 (Deformable ResBlock removed) yielded the lowest image quality, whereas Case 1 (**α** removed) showed the lowest semantic segmentation performance. Through this, Deformable ResBlock was found to have a large effect on WRA-Net’s ability to restore perceptually pleasing images and **α** to have a large effect on its ability to restore detailed features required for semantic segmentation. In Cases 4 to 7 (adjusted numbers of mDSCB groups and modules in each group), Case 4, which has the smallest number of mDSCB groups, modules per group, and thus the smallest receptive field, yielded the highest image quality following the proposed method. However, the semantic segmentation performance improved as the number of mDSCB groups and the number of mDSCB modules of each group increased. Thus, because the proposed method (Case 8) has a large receptive field and fuses the features with various receptive fields, it sufficiently restores the image to obtain excellent image quality and semantic segmentation performance.

**Table 1.** Comparisons of image quality according to the main modules of WRA-Net.

Methods	*PSNR*	*SSIM*
Case 1	30.7167	0.8835
Case 2	30.9280	0.8859
Case 3	30.0654	0.8618
Case 4	30.6706	0.8828
Case 5	30.5392	0.8732
Case 6	30.5299	0.8871
Case 7	30.4249	0.8727
Case 8 (proposed)	**31.0664**	**0.8863**

**Table 2.** Comparative accuracies of semantic segmentation according to the main modules of WRA-Net.

Methods	*mIOU*	Crop *IOU*	Weed *IOU*	BG *IOU*	*Recall*	*Precision*	*F*1 *score*
Case 1	0.7103	0.4782	0.6714	0.9814	0.7846	0.8502	0.8155
Case 2	0.7423	0.5547	0.6883	**0.9838**	0.8322	0.8428	0.8371
Case 3	0.7160	0.5134	0.6567	0.9778	0.8041	0.8375	0.8295
Case 4	0.7459	0.5627	0.6937	0.9813	0.8192	0.8727	0.8438
Case 5	0.7633	0.5859	0.7228	0.9812	0.8412	0.8780	0.8523
Case 6	0.7560	0.5797	0.7057	0.9828	0.8418	0.8618	0.8507
Case 7	0.7703	0.6138	0.7142	0.9822	0.8568	0.8643	0.8598
Case 8 (proposed)	**0.7741**	**0.6247**	**0.7143**	0.9833	**0.8596**	**0.8775**	**0.8677**

Second, an experiment was conducted based on the application of motion blur, the schemes of which are specifically described as follows.

**Scheme 1**: Train U-Net with original data without motion blur and then measure performance for the original test set.

**Scheme 2**: Train U-Net with original data without motion blur and then measure performance for motion-blurred test set.

**Scheme 3**: Train U-Net with motion-blurred data and then measure performance for motion-blurred test set.

**Scheme 4**: Train U-Net with original data without motion blur and then measure performance for the test set restored by WRA-Net.

**Scheme 5**: Train U-Net with data restored by WRA-Net and then measure performance for restored test set.

These schemes are briefly listed in Table S7.

Table 3 shows the experimental results. As evidenced by a comparison of Schemes 1 and 2, the segmentation accuracy is considerably lower when motion blur occurs than when using the original images. Also, according to the results of Schemes 2 and 3, detailed features of the crops and weeds are lost when motion blur occurs, making it difficult to distinguish between the crop, weed, and background regions and degrading the semantic segmentation accuracy. Schemes 4 and 5 provide experimental results using the images restored through WRA-Net proposed here, which showed better segmentation accuracy for all metrics than Schemes 2 and 3, which used blurred images. For Schemes 2 and 4, training was performed identically using the original data; the blurred image was restored in Scheme 4, yielding a 0.11 higher *mIOU* than Scheme 2. In Scheme 5, training was performed using the restored image, achieving a similar performance (*F*1 *score*) to Scheme 1. Hence, WRA-Net demonstrates excellent performance that restores the detailed features of crops and weeds lost due to motion blur close to the original images and effectively improves the performance of crop and weed semantic segmentation reduced by motion blur.

**Table 3.** Comparison of semantic segmentation accuracies according to the application of motion blur and restoration.

Methods	*mIOU*	Crop *IOU*	Weed *IOU*	BG *IOU*	*Recall*	*Precision*	*F*1 *score*
Scheme 1	0.8040	0.6732	0.7506	0.9880	0.8819	0.8934	0.8867
Scheme 2	0.6264	0.3781	0.5356	0.9655	0.7600	0.7653	0.7615
Scheme 3	0.6786	0.4647	0.5984	0.9726	0.7874	0.7985	0.7920
Scheme 4	0.7373	0.5529	0.6759	0.9831	0.8284	0.8566	0.8412
Scheme 5	0.7741	0.6247	0.7143	0.9833	0.8596	0.8775	0.8677

The third experiment concerns the application of instance normalization in the encoder and decoder of Fig. 2. Table 4 shows the experimental results. The findings indicate that applying instance normalization positively impacts image quality and semantic segmentation, but applying it only to the encoder yields the best performance.

**Table 4.** Comparison of image quality and semantic segmentation accuracy with and without instance normalization applied to the proposed WRA-Net. “Instance” indicates that instance normalization is applied, and “batch” indicates that batch normalization is applied.

Encoder	Decoder	Image quality		Semantic segmentation accuracy
		*PSNR*	*SSIM*	*mIOU*
Batch	Batch	27.6060	0.7783	0.6300
Instance	Instance	29.4348	0.8713	0.7067
Instance	Batch	**31.0664**	**0.8863**	**0.7741**

##### Comparisons by proposed and state-of-the-art methods

This subsection compares state-of-the-art deblurring methods with the proposed WRA-Net. In Table 5, *PSNR* and *SSIM* are measured, and quantitative evaluations of the restored image quality for the proposed WRA-Net and state-of-the-art methods are presented. WRA-Net achieved the highest values for both *PSNR* and *SSIM*.

**Table 5.** Comparisons of image quality by proposed and state-of-the-art methods. “No restoration” indicates the similarity measurement between the blurred images without restoration and the original images.

Methods	*PSNR*	*SSIM*
No restoration	24.9922	0.7090
DeblurGANv2 [57]	27.7101	0.8411
HINet [31]	28.1810	0.8006
MIMO-UNet [53]	29.0000	0.8100
MPRNet [58]	26.4485	0.7367
NAFNet [59]	28.3807	0.8122
WRA-Net (proposed)	**31.0664**	**0.8863**

**Fig. 3.**
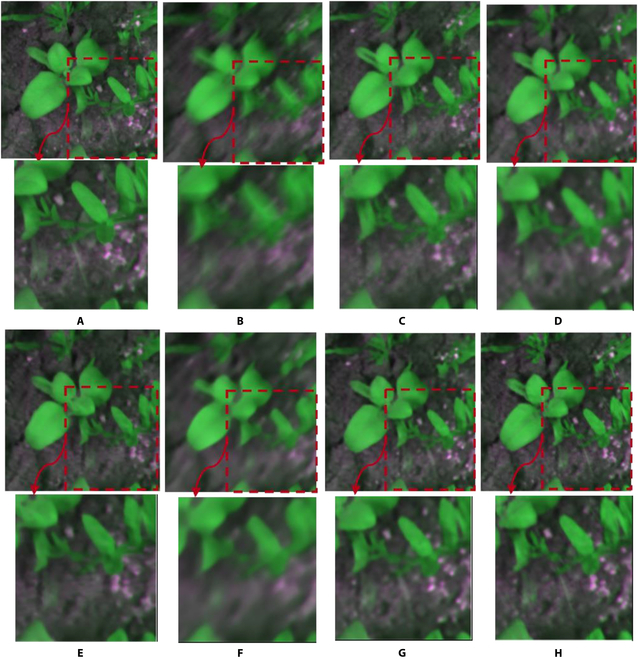
Examples of restored images by the state-of-the-art methods and the proposed WRA-Net. The area indicated by the red dashed box is enlarged and shown below each image. (A) Original image; (B) blurred image; (C) DeblurGANv2; (D) HINet; (E) MIMO-UNet; (F) MPRNet; (G) NAFNet; (H) WRA-Net (proposed method).

Figure 3 presents an example of a crop and weed image restored through state-of-the-art methods and WRA-Net. Examining the enlarged images in Fig. 3, the image restored by WRA-Net best expressed the details of the original image.

**Fig. 4.**
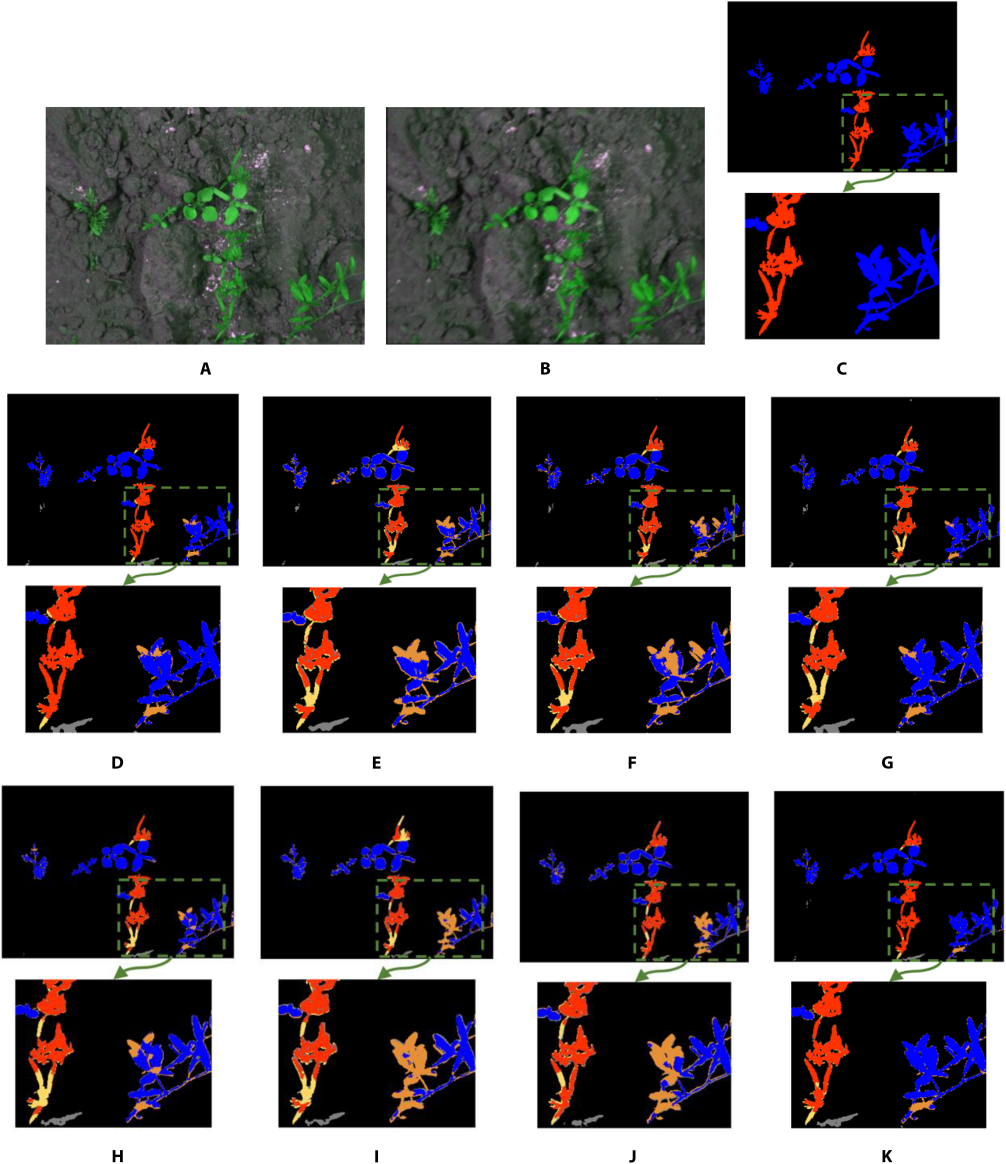
Examples of semantic segmentation results of restored images by the state-of-the-art methods and the proposed WRA-Net. The area indicated by the green dashed box is enlarged and shown below each image. Red pixels indicate crops, blue pixels indicate weeds, and black pixels indicate the background. Yellow indicates error pixels where crops were incorrectly detected as weeds or background; orange indicates error pixels where weeds were incorrectly detected as crops or background. Gray indicates error pixels where the background was incorrectly detected as crops or weeds. (A) Original image; (B) motion-blurred image; (C) ground truth label; semantic segmentation results with (D) original image, (E) blurred image, and the restored images by (F) DeblurGANv2, (G) HINet, (H) MIMO-UNet, (I) MPRNet, (J) NAFNet, and (K) WRA-Net (proposed method).

However, as the main purpose of motion blur restoration here is to improve the semantic segmentation accuracy rather than image quality, in Table 6, the performance was measured from the perspective of semantic segmentation accuracy. Table 6 shows the comparison results of crop and weed semantic segmentation performance for data with motion blur restored using the proposed WRA-Net and the state-of-the-art restoration methods. According to the results, WRA-Net yielded the highest performance in all measured metrics. Figure 4 visually compares the semantic segmentation results. All ground truth labels are weed except for the background of the enlarged part in Fig. 4.

**Table 6.** Comparative accuracies of semantic segmentation with the restored images by the state-of-the-art methods and the proposed WRA-Net. “No restoration” indicates the performance of training with blurred images without restoration.

Methods	*mIOU*	Crop *IOU*	Weed *IOU*	BG *IOU*	*Recall*	*Precision*	*F*1 *score*
No restoration	0.6786	0.4647	0.5984	0.9726	0.7874	0.7985	0.7920
DeblurGANv2 [57]	0.7029	0.4892	0.6409	0.9788	0.8043	0.8268	0.8145
HINet [31]	0.7109	0.5010	0.6513	0.9804	0.8063	0.8399	0.8215
MIMO-UNet [53]	0.7338	0.5321	0.6885	0.9806	0.8236	0.8525	0.8366
MPRNet [58]	0.6408	0.3964	0.5559	0.9701	0.7390	0.7974	0.7658
NAFNet [59]	0.7222	0.5319	0.6539	0.9806	0.8241	0.8313	0.8271
WRA-Net (proposed)	**0.7741**	**0.6247**	**0.7143**	**0.9833**	**0.8596**	**0.8775**	**0.8677**

Regarding the semantic segmentation results in the enlarged regions of the images restored by the state-of-the-art methods, compared to the proposed WRA-Net, it is visually evident that many regions were incorrectly identified as a crop. Hence, WRA-Net achieved higher values for *PSNR*and *SSIM* compared to the state-of-the-art methods, and the highest performance for semantic segmentation accuracy. This indicates that WRA-Net is the most suitable model for restoring motion-blurred images in crop and weed data and the most suitable model for systems that perform motion-blur restoration and semantic segmentation.

#### Testing with the BoniRob dataset

##### Ablation studies

In this subsection, an ablation study was conducted according to the application of motion blur to the BoniRob dataset through the same schemes as in the Ablation studies subsection under the Testing with the CWFID dataset section. Table S8 shows the results. Similar to the results in the Ablation studies subsection under the Testing with the CWFID dataset section, the segmentation accuracy was greatly reduced in Schemes 2 and 3 compared to Scheme 1 due to motion blur. The segmentation accuracy, which decreased due to motion blur, was improved by restoring the motion-blurred images through WRA-Net in Schemes 4 and 5. In addition, in *mIOU*, Scheme 5 was most similar to Scheme 1, the performance for the original data. Hence, in the BoniRob dataset, WRA-Net restores the detailed features of crops and weeds lost owing to motion blur close to the original images and improves the performance of crop and weed semantic segmentation that was reduced by motion blur.

##### Comparisons by proposed and state-of-the-art methods

This subsection compares the state-of-the-art methods and the proposed WRA-Net for the BoniRob dataset through the same method as the Comparisons by proposed and state-of-the-art methods section. In Table 7, *PSNR* and *SSIM* are measured, and qualitative evaluations of the motion-blurred image restoration quality for the proposed WRA-Net and state-of-the-art methods are presented. *PSNR* was the highest for MIMO-UNet [53], whereas *SSIM* was the highest for WRA-Net.

**Table 7.** Comparisons of image quality by proposed and state-of-the-art methods. “No restoration” indicates the similarity measurement between the blurred images without restoration and the original images.

Models	*PSNR*	*SSIM*
No restoration	27.4502	0.8342
DeblurGANv2 [57]	33.3309	0.7596
HINet [31]	38.7071	0.9656
MIMO-UNet [53]	**42.6200**	0.9700
MPRNet [58]	36.8939	0.9438
NAFNet [59]	38.0399	0.9620
WRA-Net (proposed)	41.5979	**0.9731**

**Fig. 5.**
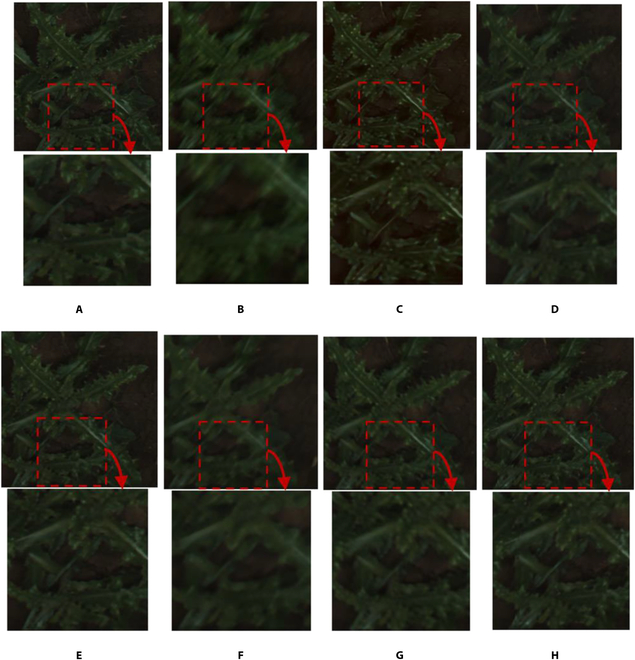
Examples of restored images by the state-of-the-art methods and the proposed WRA-Net. The area indicated by the red dashed box is enlarged and shown below each image. (A) Original image; (B) blurred image; (C) DeblurGANv2; (D) HINet; (E) MIMO-UNet; (F) MPRNet; (G) NAFNet; (H) WRA-Net (proposed method).

**Fig. 6.**
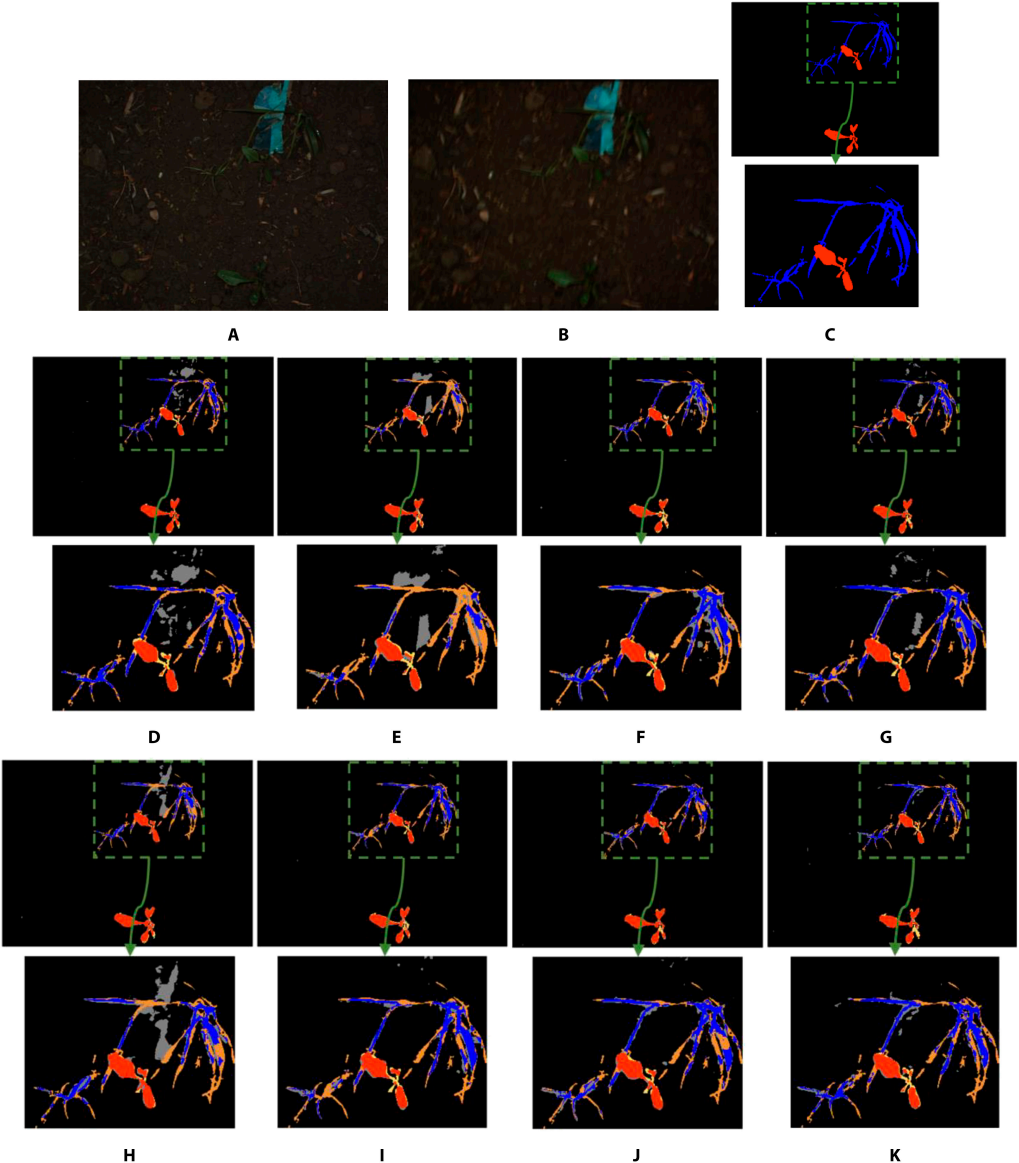
Examples of semantic segmentation results of restored images by the state-of-the-art methods and the proposed WRA-Net. The area indicated by the green dashed box is enlarged and shown below each image. Red pixels indicate crops, blue pixels indicate weeds, and black pixels indicate the background. Yellow indicates error pixels where crops were incorrectly detected as weeds or background; orange indicates error pixels where weeds were incorrectly detected as crops or background. Gray indicates error pixels where the background was incorrectly detected as crops or weeds. (A) Original image; (B) motion-blurred image; (C) ground truth label; semantic segmentation results with (D) original image, (E) blurred image, and the restored images by (F) DeblurGANv2, (G) HINet, (H) MIMO-UNet, (I) MPRNet, (J) NAFNet, and (K) WRA-Net (proposed method).

Figure 5 presents an example of an image restored through state-of-the-art methods and WRA-Net. This demonstrates that WRA-Net successfully restored the motion-blurred crop and weed image close to the original image. However, as explained in the Comparisons by proposed and state-of-the-art methods section, the main purpose of motion-blurred image restoration here is to improve the crop and weed semantic segmentation performance, not to improve image quality. As such, the semantic segmentation results were evaluated in Table 8 and visualized in Fig. 6. As shown in Table 8, WRA-Net yielded better segmentation accuracy than the state-of-the-art methods in all metrics. Comparing the segmentation results for the area within the green dashed box in Fig. 6, WRA-Net obtained the most accurate results. In particular, in Fig. 6E, G, and H, the vinyl area included in the background class was incorrectly classified as crop class, and it was visualized as error pixels in the figure (gray pixels). However, as shown in Fig. 6K, the semantic segmentation result of the image restored by the proposed WRA-Net showed that the vinyl area was well segmented as background class. This confirms that WRA-Net is good at reconstructing detailed features from motion-blurred images so that objects are well distinguished. As a result, even the very thin weed region shown in the bottom-left area within the dashed box, which is difficult to be discriminated, showed better segmentation results than those by the other state-of-the-art models.

**Table 8.** Comparative accuracies of semantic segmentation with the restored images by the state-of-the-art methods and the proposed WRA-Net. “No restoration” indicates the performance of training with blurred images without restoration.

Models	*mIOU*	Crop *IOU*	Weed *IOU*	BG *IOU*	*Recall*	*Precision*	*F*1 *score*
No restoration	0.7084	0.7978	0.3471	0.9802	0.7798	0.8169	0.7970
DeblurGANv2 [57]	0.7003	0.7984	0.3230	0.9795	0.7693	0.8099	0.7878
HINet [31]	0.7382	0.8192	0.4124	0.9831	0.8138	0.8391	0.8250
MIMO-UNet [53]	0.7307	0.8187	0.3912	0.9823	0.8047	0.8277	0.8151
MPRNet [58]	0.7179	0.8004	0.3728	0.9806	0.7935	0.8166	0.8042
NAFNet [59]	0.7380	0.8164	0.4144	0.9832	0.8115	0.8375	0.8235
WRA-Net (proposed)	**0.7444**	**0.8269**	**0.4224**	**0.9838**	**0.8165**	**0.8420**	**0.8282**

#### Testing with rice seedling and weed dataset

##### Ablation studies

This subsection performed an ablation study concerning the application of motion blur to the rice seedling and weed dataset through the same method as the Ablation studies subsection under the Testing with the CWFID dataset section and the Ablation studies subsection under the Testing with the BoniRob dataset section. The results are shown in Table S9. Similar to the results in the Ablation studies subsection under the Testing with the CWFID dataset section and the Ablation studies subsection under the Testing with the BoniRob dataset section, the recognition accuracy that degraded owing to motion blur in Schemes 2 and 3 was improved by restoring the motion-blurred images through WRA-Net in Schemes 4 and 5. In addition, Scheme 5 yielded the closest semantic segmentation accuracy to Scheme 1, which is the result for the original data. Hence, in the rice seedling and weed dataset, WRA-Net restores the detailed features of crops and weeds lost owing to motion blur close to the original images and improves the performance of crop and weed semantic segmentation that was reduced by motion blur.

##### Comparisons by proposed and state-of-the-art methods

**Fig. 7.**
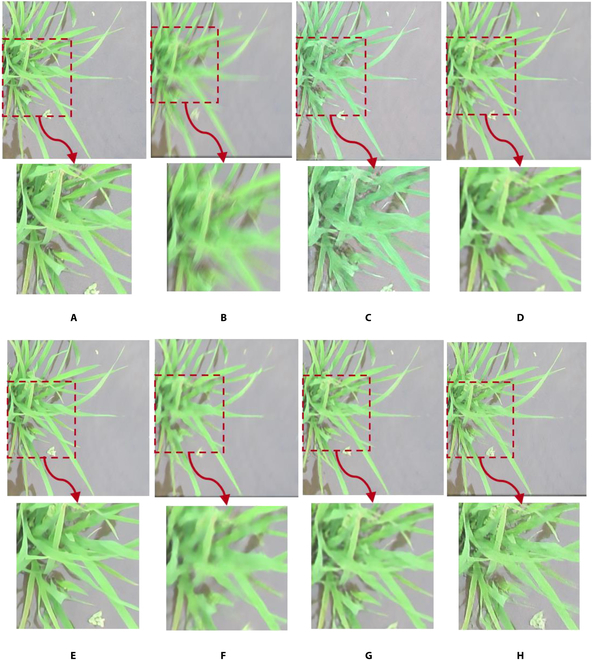
Examples of restored images by state-of-the-art methods and the proposed WRA-Net. The area indicated by the red dashed box is enlarged and shown below each image. (A) Original image; (B) blurred image; (C) DeblurGANv2; (D) HINet; (E) MIMO-UNet; (F) MPRNet; (G) NAFNet; and (H) WRA-Net (proposed method).

**Fig. 8.**
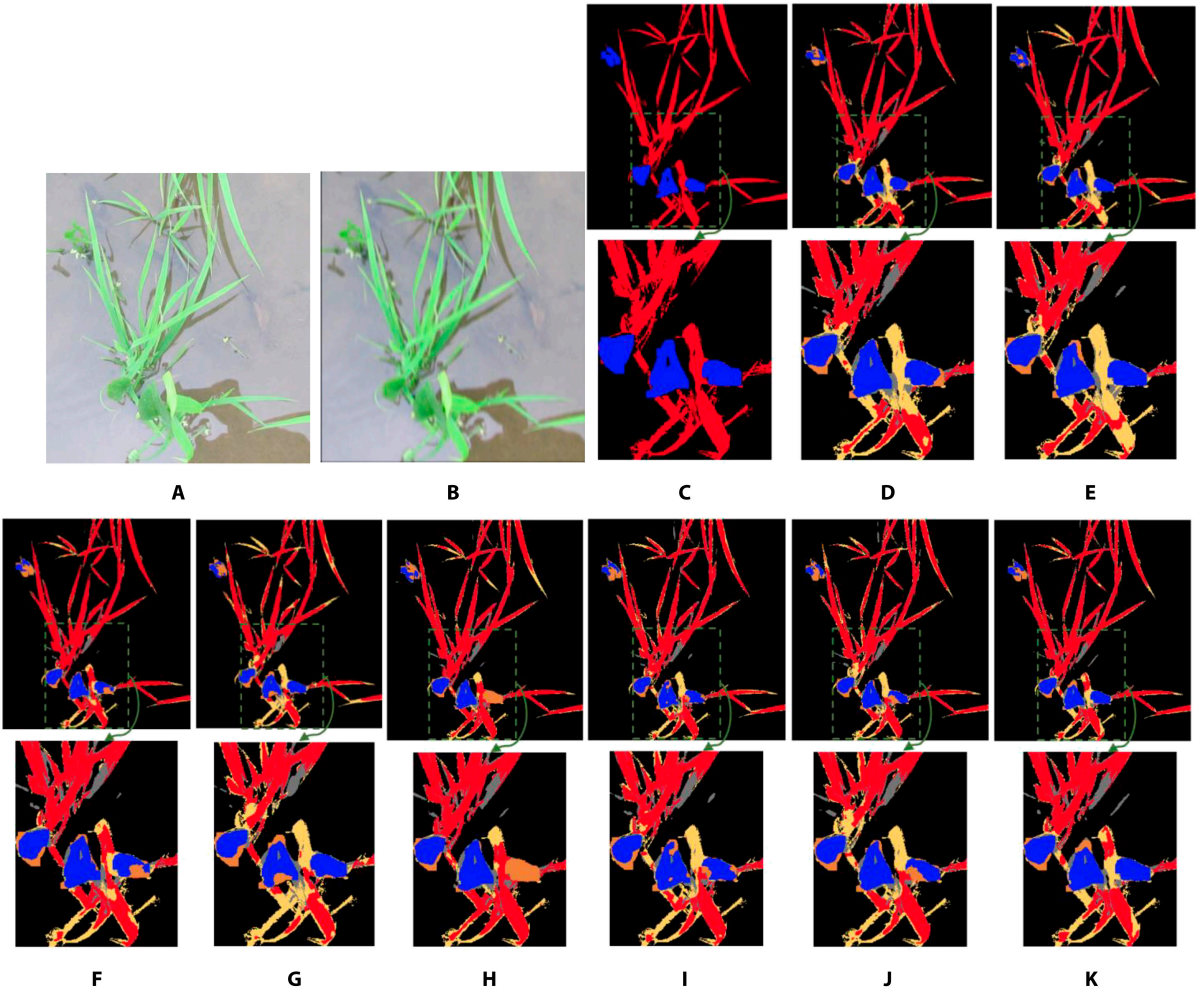
Examples of semantic segmentation results of restored images by the state-of-the-art methods and the proposed WRA-Net. The area indicated by the green dashed box is enlarged and shown below each image. Red pixels indicate crops, blue pixels indicate weeds, and black pixels indicate the background. Yellow indicates error pixels where crops were incorrectly detected as weeds or background; orange indicates error pixels where weeds were incorrectly detected as crops or background. Gray indicates error pixels where the background was incorrectly detected as crops or weeds. (A) original image; (B) motion-blurred image; (C) ground truth label; semantic segmentation results with (D) original image, (E) blurred image, and the restored images by (F) DeblurGANv2, (G) HINet, (H) MIMO-UNet, (I) MPRNet, (J) NAFNet, and (K) WRA-Net (proposed method).

This subsection compares the state-of-the-art methods and the proposed WRA-Net for the rice seedling and weed dataset through the same method as in the Comparisons by proposed and state-of-the-art methods section and the Ablation studies subsection under the Testing with the BoniRob dataset section. In Table 9, *PSNR* and *SSIM* are measured, and quantitative evaluations of the motion-blurred image restoration quality for the proposed WRA-Net and state-of-the-art methods are presented. Figure 7 presents an example of an image from the rice seedling and weed dataset restored through state-of-the-art methods and WRA-Net. According to the image quality metrics in Table 9, *PSNR* was the highest for MIMO-UNet [55], whereas *SSIM* was the highest for WRA-Net. As evidenced in Fig. 7, WRA-Net restored the image close to the original. As mentioned in the Comparisons by proposed and state-of-the-art methods section and the Ablation studies subsection under the Testing with the BoniRob dataset section, because this study aims to improve semantic segmentation performance rather than image quality, the semantic segmentation results are evaluated in Table 10 and visualized in Fig. 8. As shown in Table 10, WRA-Net yielded better performance than the state-of-the-art methods in all semantic segmentation metrics except *Precision*. Figure 8 visualizes these results. Comparing the segmentation results for the area within the green dashed box, WRA-Net obtained the most accurate results.

**Table 9.** Comparisons of image quality by proposed and state-of-the-art methods. “No restoration” indicates the similarity measurement between the blurred images without restoration and the original images.

Models	*PSNR*	*SSIM*
No restoration	16.9639	0.5397
DeblurGANv2 [57]	25.9088	0.8054
HINet [31]	28.0917	0.8956
MIMO-UNet [53]	**30.1800**	0.9000
MPRNet [58]	26.1525	0.8061
NAFNet [59]	29.1002	0.8929
WRA-Net (proposed)	29.1871	**0.9031**

**Table 10.** Comparative accuracies of semantic segmentation with the restored images by state-of-the-art methods and the proposed WRA-Net. “No restoration” indicates the performance of training with blurred images without restoration.

Models	*mIOU*	Crop *IOU*	Weed *IOU*	BG *IOU*	*Recall*	*Precision*	*F*1 *score*
No restoration	0.6911	0.5758	0.5722	0.9253	0.8210	0.7983	0.8078
DeblurGANv2 [57]	0.7046	0.6096	0.5727	0.9315	0.8263	0.8185	0.8202
HINet [31]	0.6995	0.5840	0.5861	0.9284	0.8193	0.8141	0.8149
MIMO-UNet [53]	0.7085	0.6194	0.5734	0.9326	0.8337	**0.8185**	0.8237
MPRNet [58]	0.6911	0.5791	0.5691	0.9252	0.8118	0.8089	0.8086
NAFNet [59]	0.7038	0.5958	0.5860	0.9295	0.8225	0.8157	0.8174
WRA-Net (proposed)	**0.7149**	**0.6236**	**0.5873**	**0.9340**	**0.8407**	0.8153	**0.8260**

### Processing time and computational cost

This subsection measured the inference time, the number of model parameters, GPU memory requirement, floating point operations (FLOPs), and multiply-and-accumulates (MACs) of WRA-Net proposed in this study. First, the inference time in a Jetson-embedded system and the desktop computer was measured. Jetson TX2 is the embedded system with NVIDIA Pascal^TM^-family GPU, which has 256 CUDA cores and uses less than 7.5 W of power. It has 8 GB shared memory between GPU and CPU. Figure S9 shows Jetson TX2’s architecture [54]. The desktop computer specifications are described in the Experimental dataset and setup subsection. This was measured in a Jetson TX2 embedded system for the following reasons. When crop and weed segmentation is used in a farming robot, the algorithm is often executed as onboard computing on an embedded system in the robot. Therefore, to verify that the proposed system can operate in the embedded system, we performed the experiment on a Jetson TX2 embedded system, the results of which are shown in Table S10. The inference time was measured in 2 cases for a more detailed comparison. First, the inference time of the WRA-Net restoration process for the motion-blurred image was measured. Second, the inference time of the semantic segmentation process through U-Net after restoring the motion-blurred image with WRA-Net was measured. In the first case, the inference time for one image was 0.1850 s on the desktop computer and 1.5529 s on the Jetson embedded system. In the second case, the inference time for one image was 0.2468 s on the desktop computer and 2.3430 s on the Jetson embedded system. These results indicate that the method proposed here can be operated even in an embedded system with limited computing resources.

Table S11 compares the number of parameters, GPU memory requirements, FLOPs, and MACs of the proposed WRA-Net and state-of-the-art methods. Furthermore, to relatively evaluate the computational cost, Fig. S10 compares the segmentation accuracy vs. MACs, GPU memory consumption per image, the number of parameters, and FLOPs between the proposed method and state-of-the-art models.

Compared to the state-of-the-art methods, the proposed WRA-Net shows that the number of parameters is the smallest and MACs and FLOPs are more than other methods, but smaller than MPRNet and HINet. The GPU memory requirement of WRA-Net is the third smallest after NAFNet and DeblurGANv2. Nevertheless, as shown in Tables 6, 8, and 10 and Fig. S10, the proposed method yielded the highest segmentation accuracy.

## Discussion

**Fig. 9.**
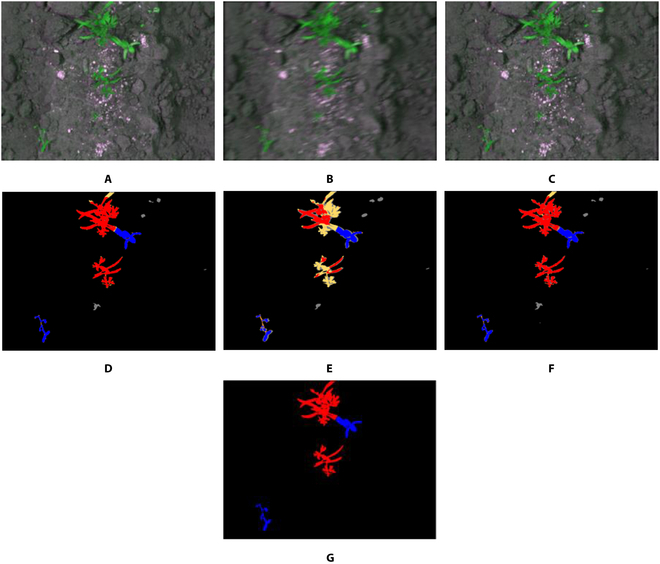
An example of correctly segmented case by our method. Red pixels indicate crops, blue pixels indicate weeds, and black pixels indicate the background. Yellow indicates error pixels where crops were incorrectly detected as weeds or background; orange indicates error pixels where weeds were incorrectly detected as crops or background. Gray indicates error pixels where the background was incorrectly detected as crops or weeds. (A) Original image, (B) motion-blurred image, (C) restored image by WRA-Net. Semantic segmentation results with (D) original image, (E) blurred image, and (F) restored image by WRA-Net; (G) ground truth label.

In this subsection, we analyze semantic segmentation results with correctly and incorrectly segmented cases, and gradient-weighted class activation mapping (Grad-CAM) [55,56]. Figure 9 shows an example of a correctly segmented case by our method. In case of severe motion-blurred image as shown in Fig. 9B, semantic segmentation error occurs in a large area. When performing semantic segmentation after restoring motion-blurred images as shown in Fig. 9C by WRA-Net, error pixels are greatly reduced as shown in Fig. 9F.

**Fig. 10.**
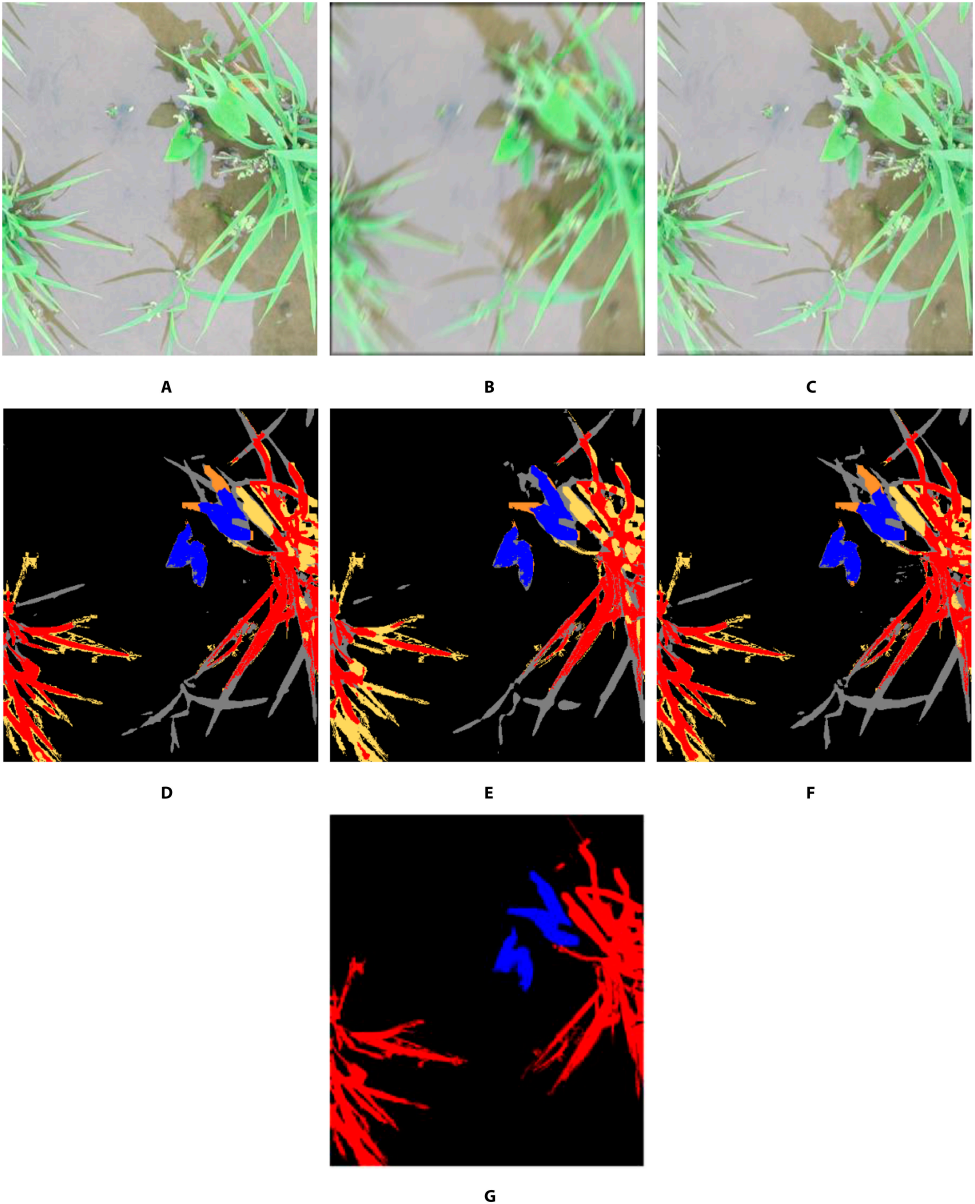
An example of incorrectly segmented case despite restoring motion-blurred image by WRA-Net. Red pixels indicate crops, blue pixels indicate weeds, and black pixels indicate the background. Yellow indicates error pixels where crops were incorrectly detected as weeds or background; orange indicates error pixels where weeds were incorrectly detected as crops or background. Gray indicates error pixels where the background was incorrectly detected as crops or weeds. (A) Original image, (B) motion-blurred image, and (C) restored image by WRA-Net. Semantic segmentation results with (D) original image, (E) blurred image, and (F) restored image by WRA-Net; (G) ground truth label.

Figure 10 shows the example of incorrectly segmented case despite restoring motion-blurred image by WRA-Net. As shown in Fig. 10B, even though the image with motion blur was restored by WRA-Net as shown in Fig. 10C, the semantic segmentation result was not greatly improved as shown in Fig. 10F. We estimated that it is because even though detailed features were restored by WRA-Net, it is difficult to segment correctly when weed and crop have similar shapes and colors, and objects have thin regions as in this example.

Figure S8 shows the result of analyzing the semantic segmentation result with Grad-CAM. In the original image, motion-blurred image, and restored image by WRA-Net, we visualize the area where U-Net focuses in each class. In the case of the motion-blurred image, class activation does not appear in the exact area corresponding to each class, but class activation appears in other wide areas. In the case of restored image by WRA-Net, class activation appears within the exact area corresponding to each class, which shows a similar pattern to class activation in the original image. This confirms that the restoration of motion-blurred image by WRA-Net improves crop and weed segmentation results.

This study investigated a method to improve crop and weed semantic segmentation performance by restoring motion-blurred crop and weed images to solve the problem of performance degradation in crop and weed semantic segmentation due to motion blur. The proposed WRA-Net is a motion blur restoration model optimized for crop and weed images. WRA-Net receives motion-blurred images as input, integrates features of various receptive fields through Lite WRARB in the encoder, extracts features useful for motion blur restoration, and passes them to the decoder. The decoder samples the features received from the encoder in a flexible receptive field through Deformable ResBlock, restores the motion blur, and outputs images with restored detailed features needed for semantic segmentation. The ablation study results in the Ablation studies subsection under the Testing with the CWFID dataset section demonstrated that Lite WRARB and Deformable ResBlock improve the restoration performance for motion-blurred images. In experiments with 3 datasets, the method proposed here yielded higher semantic segmentation metrics than before restoration when performing crop and weed semantic segmentation. According to the results of experiments comparing various state-of-the-art restoration models with the proposed WRA-Net, when the latter was applied, the detailed segmentation features lost due to motion blur were accurately restored, and the highest segmentation performance (*mIOU*) was achieved. The *mIOU*s of test images restored by the proposed WRA-Net are 0.7741, 0.7444, and 0.7749, respectively, in the CWFID, BoniRob, and rice seedling and weed database.

Moreover, the experimental results in the Processing time and computational cost subsection demonstrated that the proposed method operates normally in an embedded system. However, even with the proposed restoration method, the segmentation performance after motion blur restoration was lower than that when using the original image. Because 2 stages of restoration and segmentation are required, the inference time becomes very long. Furthermore, as examined in Section 4, a segmentation error occurred when the shape and color of weed and crop were similar to each other, and the object had a thin area.

In future studies, we would research about the method using preprocessing to reduce the errors caused by the high similarity of crop and weed and the thin area of the object. In addition, we would research about the feature fusion that can obtain high semantic segmentation results directly from the motion-blurred image without performing 2 steps of restoration and semantic segmentation. Furthermore, we would research to solve various problems in crop and weed semantic segmentation, such as the lack of labeled data and the disparity in recognition performance between crops and weeds.

## Data Availability

The data supporting the findings of this study are available from the corresponding author (K.R.P.) upon request.

## Supplementary Materials

Supplementary Materials Table S1. Summarized comparisons of the proposed and related works on crop and weed segmentation. Table S2. Descriptions of 3 open datasets for training, validation, and testing. Table S3. Hyperparameter setup used for training of WRA-Net. Table S4. Hyperparameter setup used for training of U-Net. Table S5. Comparison of segmentation accuracy for blurred data and restored data of various semantic segmentation models. Table S6. Summary of cases of ablation studies for proposed modules. Table S7. Summary of schemes of ablation studies according to motion blur application and restoration. Table S8. Comparison of semantic segmentation accuracies according to the application of motion blur and restoration. Table S9. Comparison of semantic segmentation accuracies according to motion blur application and restoration. Table S10. Inference time of proposed method on desktop and Jetson embedded system. Table S11. Comparisons of number of parameters, GPU memory, FLOPs, MACs of WRA-Net, and the state-of-the-art methods. Fig. S1. Comparison of semantic segmentation results for the original image without motion blur and semantic segmentation results for image with motion blur. Fig. S2. Detailed architecture of Lite WRARB. Fig. S3. Detailed architecture of Conv Block 1, mDSCB, attention gate, and Conv Block 2. Fig. S4. Detailed architecture of decoder module. Fig. S5. Detailed architecture of convolutional block 3. Fig. S6. Samples of crop and weed public datasets. Fig. S7. Graphs of losses. Fig. S8. Comparisons of the class activation maps between the original image, motion-blurred image, and restored image. Fig. S9. Jetson TX2 embedded system. Fig. S10. Comparison of computational cost, GPU memory consumption, and semantic segmentation between state-of-the-art models and proposed method.
