# Supplementary material for: WRA-Net: Wide Receptive Field Attention Network for Motion Deblurring in Crop and Weed Image
Source: Plant Phenomics. 2023 Apr 5;5:0031. doi: 10.34133/plantphenomics.0031 (PMC10243196; doi:10.34133/plantphenomics.0031)
Supplement: Supplementary Materials — Table S1. Summarized comparisons of the proposed and related works on crop and weed segmentation. Table S2. Descriptions of 3 open datasets for training, validation, and testing. Table S3. Hyperparameter setup used for training of WRA-Net. Table S4. Hyperparameter setup used for training of U-Net. Table S5. Comparison of segmentation accuracy for blurred data and restored data of various semantic segmentation models. Table S6. Summary of cases of ablation studies for proposed modules. Table S7. Summary of schemes of ablation studies according to motion blur application and restoration. Table S8. Comparison of semantic segmentation accuracies according to the application of motion blur and restoration. Table S9. Comparison of semantic segmentation accuracies according to motion blur application and restoration. Table S10. Inference time of proposed method on desktop and Jetson embedded system. Table S11. Comparisons of number of parameters, GPU memory, FLOPs, MACs of WRA-Net, and the state-of-the-art methods. Fig. S1. Comparison of semantic segmentation results for the original image without motion blur and semantic segmentation results for image with motion blur. Fig. S2. Detailed architecture of Lite WRARB. Fig. S3. Detailed architecture of Conv Block 1, mDSCB, attention gate, and Conv Block 2. Fig. S4. Detailed architecture of decoder module. Fig. S5. Detailed architecture of convolutional block 3. Fig. S6. Samples of crop and weed public datasets. Fig. S7. Graphs of losses. Fig. S8. Comparisons of the class activation maps between the original image, motion-blurred image, and restored image. Fig. S9. Jetson TX2 embedded system. Fig. S10. Comparison of computational cost, GPU memory consumption, and semantic segmentation between state-of-the-art models and proposed method. [file plantphenomics.0031.f1.docx]

Supplementary Materials


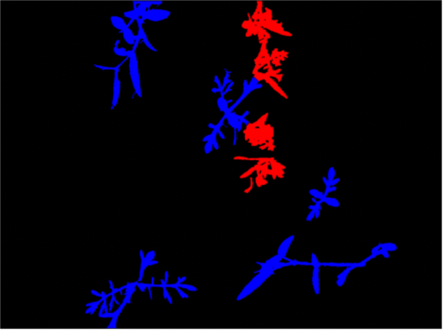


(a)


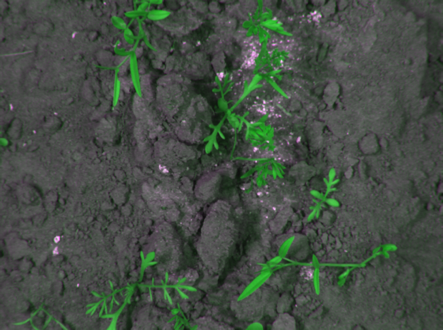

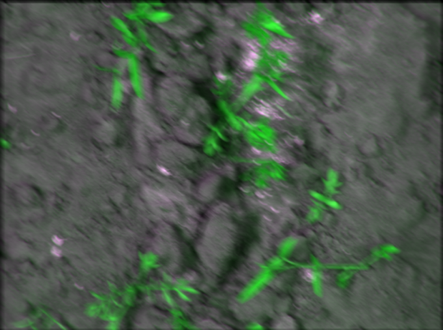


(b) (c)


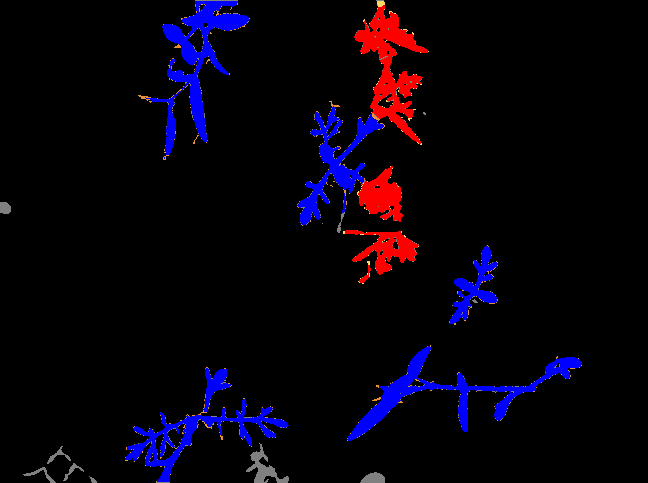

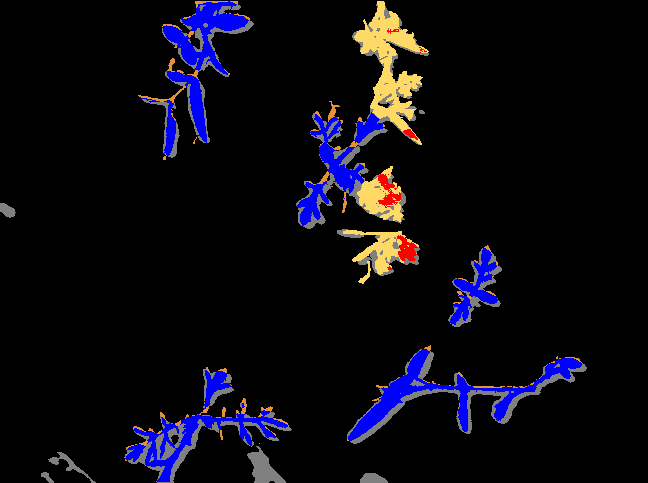


(d) (e)

Figure S1. Comparison of semantic segmentation results for the original image without motion blur and semantic segmentation results for image with motion blur. The accuracy of the segmentation results for the motion-blurred image is poor. Red indicates weeds, blue indicates crops, and black indicates the background. Yellow indicates error pixels where crops were incorrectly detected as weeds or background, orange indicates error pixels where weeds were incorrectly detected as crops or background. gray indicates error pixels where the background was incorrectly detected as crops or weeds. (a) Ground truth label; (b) original image; (c) motion-blurred image; (d) semantic segmentation result of the original image; (e) semantic segmentation result of motion-blurred image


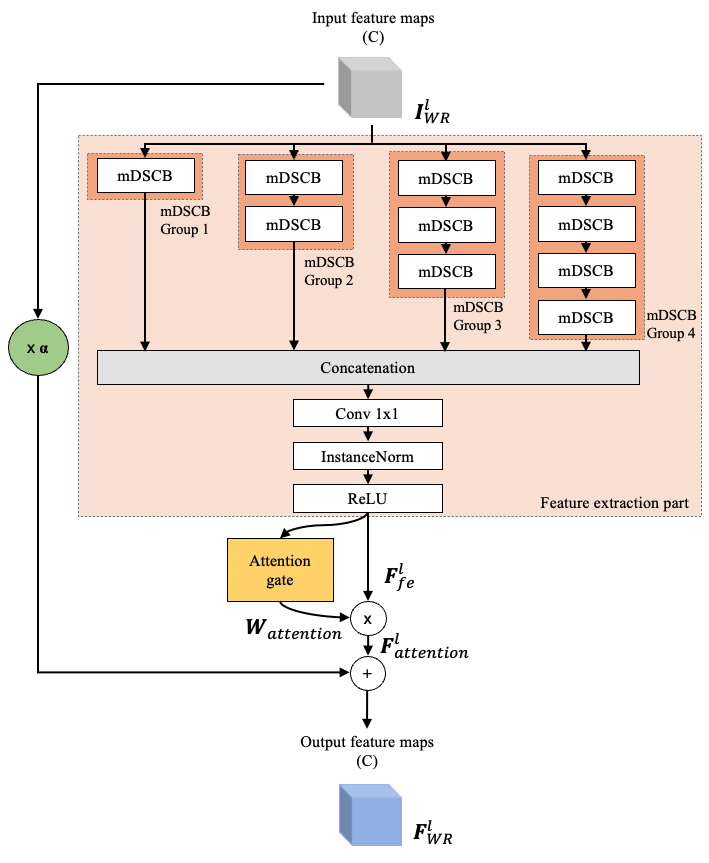


**Figure S2.** Detailed architecture of Lite WRARB.

**
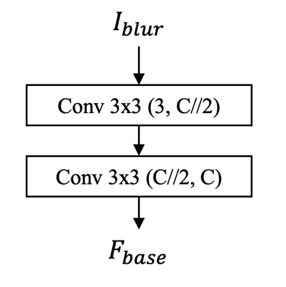

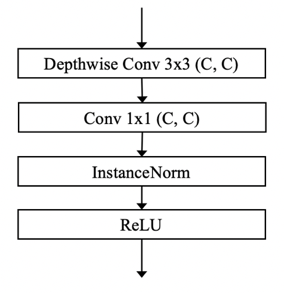
**

**(a) (b)**

**
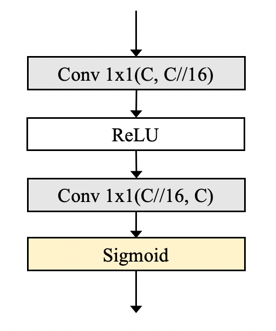

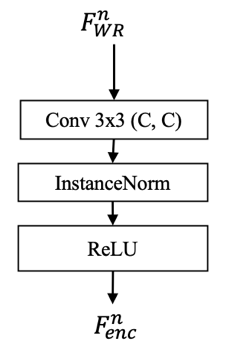
**

**(c) (d)**

**Figure S3.** Detailed architecture of Conv Block 1, mDSCB, attention gate, and Conv Block 2. The number of channels in each module’s input and output is expressed as (number of channels in input, number of channels in output). For example, (3, C//2), (C, C). C//n means the quotient of C divided by n. (a) Conv Block 1; (b) mDSCB; (c) attention gate; (d) Conv Block 2.


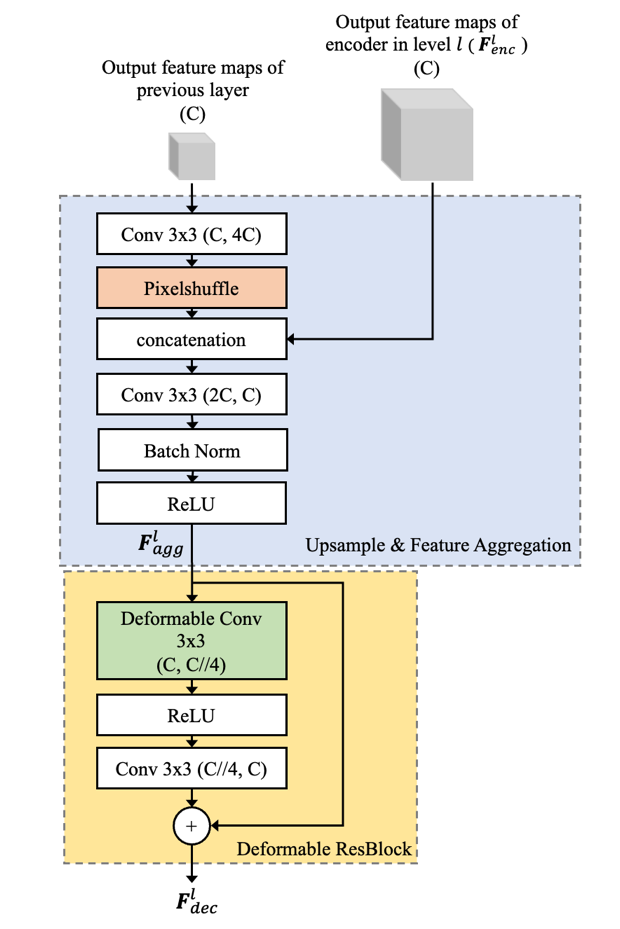


**Figure S4.** Detailed architecture of decoder module. C//n means the quotient of C divided by n.


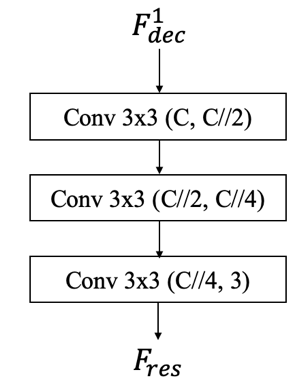


**Figure S5.** Detailed architecture of convolutional block 3 (Conv Block 3 C//n means the quotient of C divided by n.

| **Input Image** | **Ground-truth label**  **(white: crop, gray: weed)** |
| --- | --- |
| 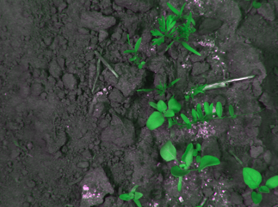 | 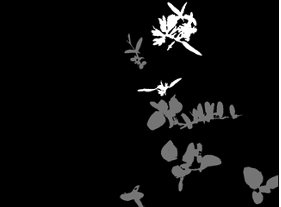 |
| **(a)** | |
| 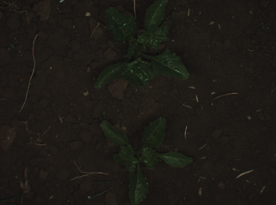 | 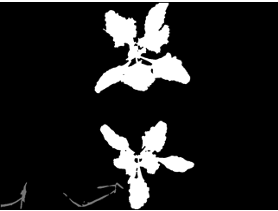 |
| **(b)** | |
| 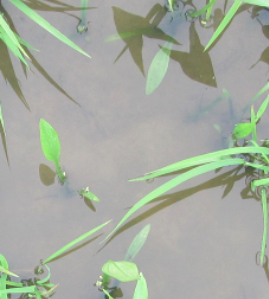 | 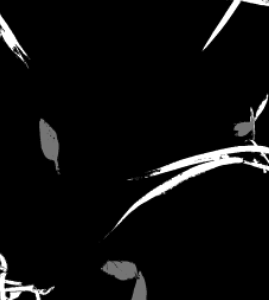 |
| **(c)** | |

Figure S6. Samples of crop and weed public datasets. (a) Crop/weed field image dataset (CWFID) dataset; (b) BoniRob dataset; (c) Rice seedling and weed dataset


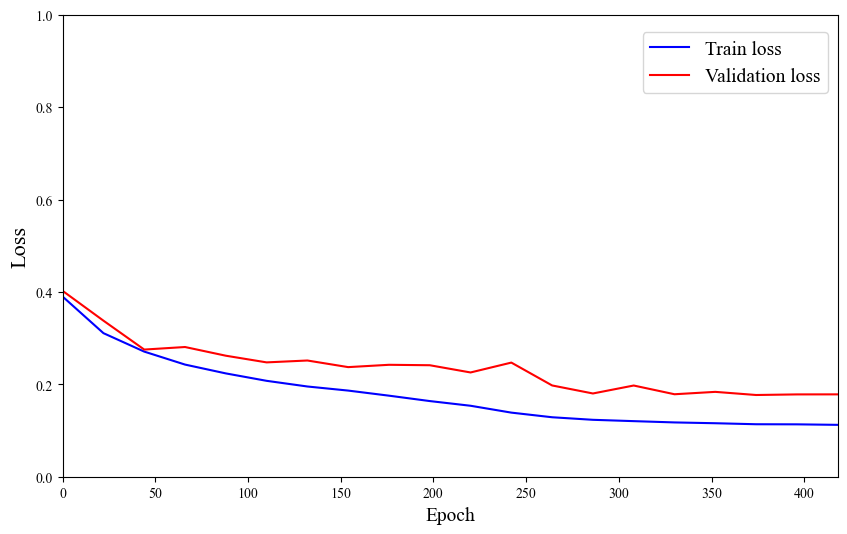


**Figure S7**. Graphs of losses. Blue and red lines represent loss graphs with training and validation data, respectively. This graph is obtained from training on CWFID database.


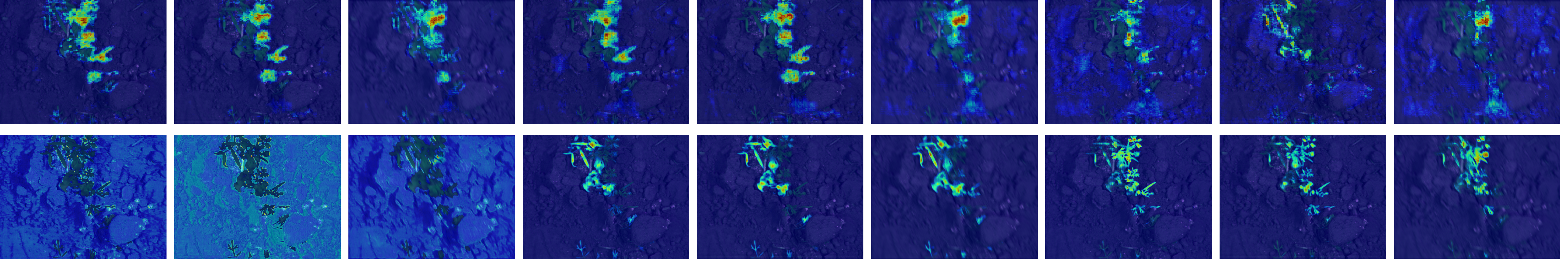


(a)


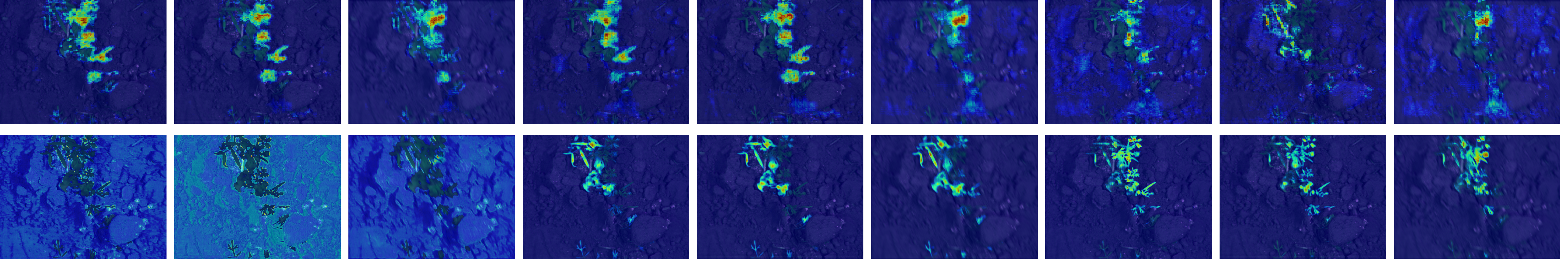


(b)


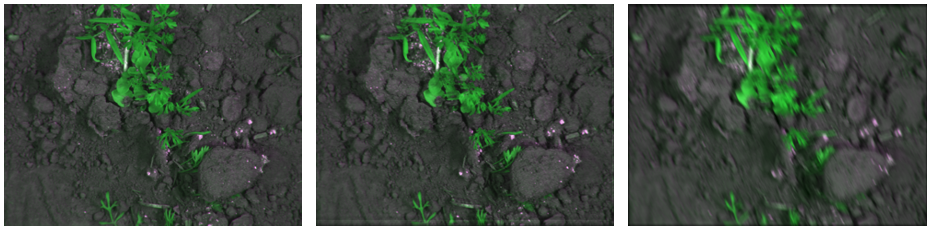


(c)

**Figure S8**. Comparisons of the class activation maps between the original image, motion-blurred image, and restored image. The 1^st^ and 2^nd^ rows of (a) and (b) show the class activation maps from the layer before the second upsampling layer and last layer of U-Net, respectively. In addition, the 1^st^, 2^nd^, and 3^rd^ columns of (a) and (b) show the class activation maps with original image, restored image by WRA-Net, and motion-blurred image. (a) and (b) show the class activation maps of crop and weed, respectively. Furthermore, the 1^st^, 2^nd^, and 3^rd^ columns of (c) are original image, restored image by WRA-Net, and motion-blurred image, respectively.


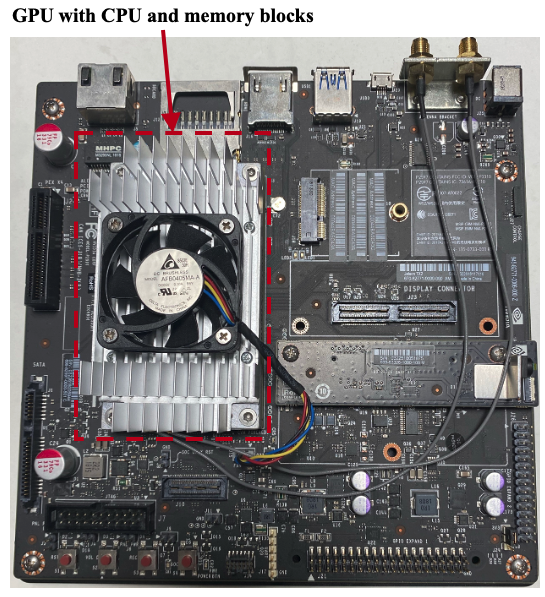


Figure S9. Jetson TX2 embedded system


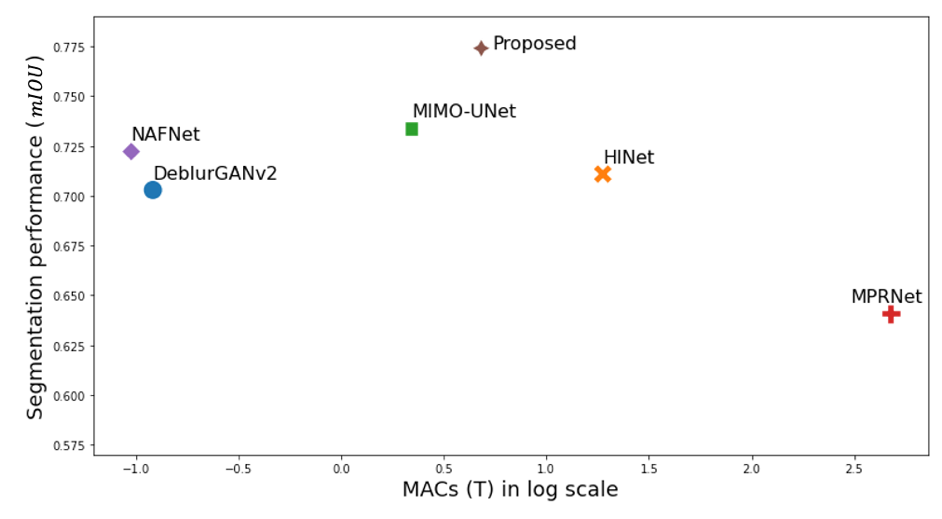

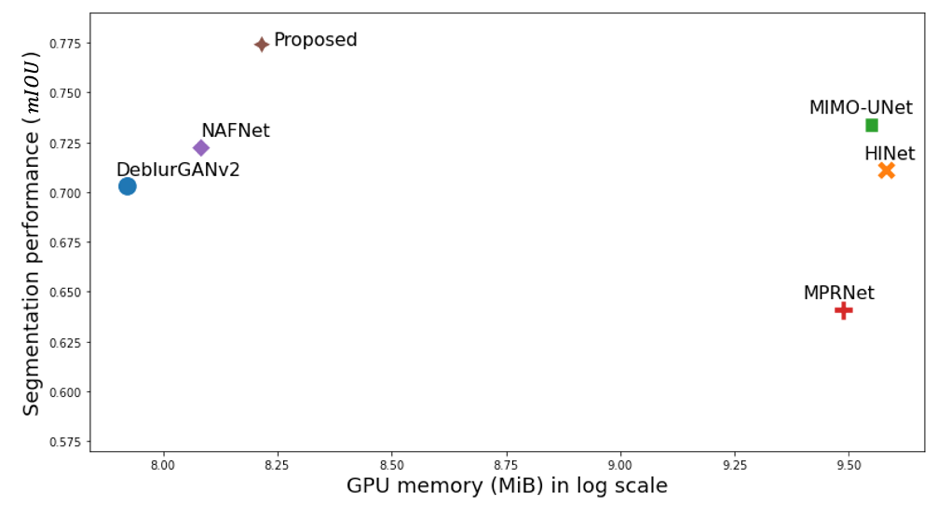


(a) (b)


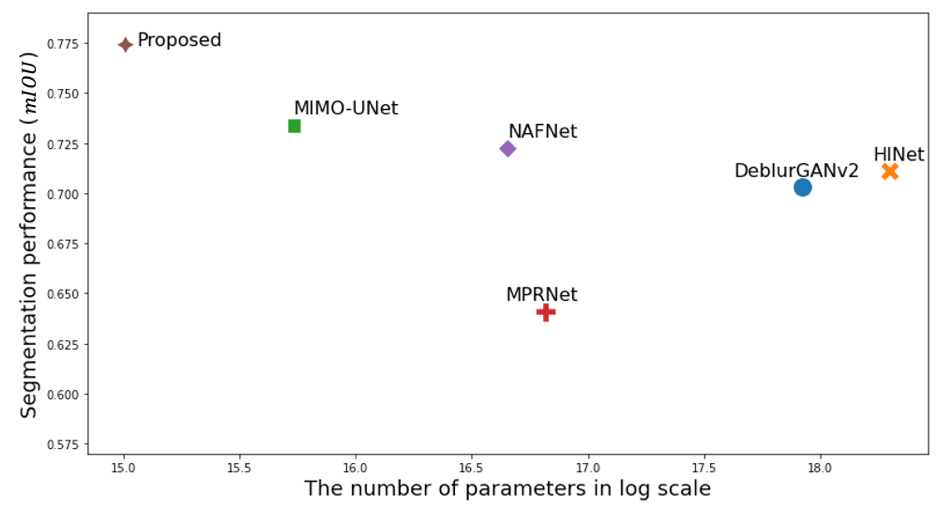
**
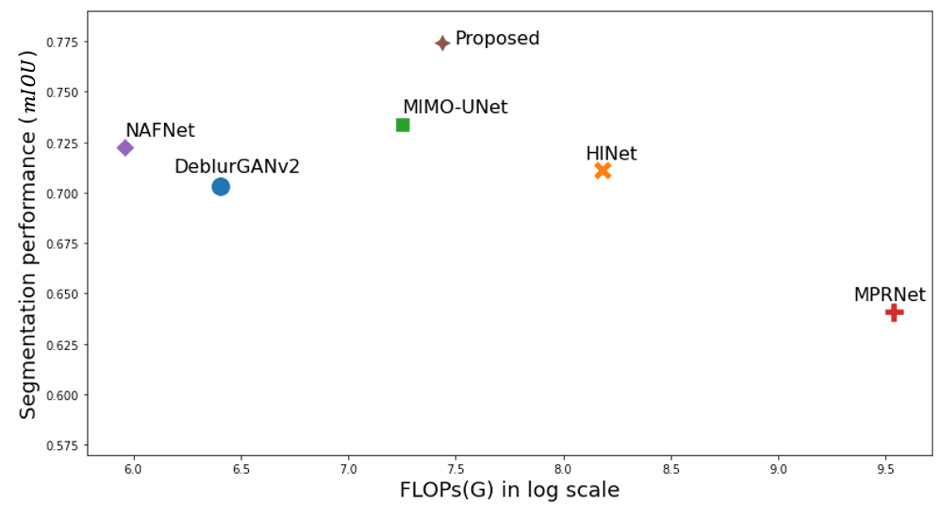
**

(c) (d)

Figure S10. Comparison of computational cost, GPU memory consumption, and semantic segmentation between state-of-the-art models and proposed method: (a) $mIOU$-MACs trade-off plot; (b) $mIOU$- GPU memory consumption trade-off plot; (c) $mIOU$-the number of parameters trade-off plot; (d) $mIOU$-FLOPs trade-off plot

**Table S1.** Summarized comparisons of the proposed and related works on crop and weed segmentation

| Category | | Method | Advantage | Disadvantage |  |
| --- | --- | --- | --- | --- | --- |
| Crop and weed segmentation  not considering motion blur | Handcrafted feature-based | Method that uses HSI color space images as input and performs Mahalanobis distance-based classification [8]. | The normalized values of the H and S channels were used as features to improve the segmentation accuracy in various light environments. | Accuracy may degrade because it does not use the G channel component, which is considered important in distinguishing crops and weeds from the background in various studies. |  |
|  |  | Method that performs random forest classification based on key point-based features or object-based features for crop and weed regions separated from the background [10]. | Because it uses NDVI images, it is not significantly impacted by various light environments. It also reduces the inference time when object-based features are used. | If the NDVI image distribution is not a bimodal intensity distribution, a binary image that separates the crop and weed region and background region may be incorrectly generated. |  |
|  |  | Method that separates crops and weeds from the background with the ExG method and Otsu threshold and then classifies the crops and weeds through SVDD [12]. | Has fast inference speed because the crops and weeds are separated from the background using only RGB channel values. | If the crop or weed color is not green, it is difficult to apply and the threshold may not be accurate. |  |
|  | Deep  feature-based | CNN model applying Enet and Segnet, which concatenate various types of images obtained from the input images and uses them as input [15] | Fourteen images obtained by transforming the input are concatenated in the channel direction and used as input, thus reducing the effect of changes in the surroundings and improving the segmentation performance. | Generating 14 channels with transformed input is time-consuming, and as the number of input channels increases, the number of convolutional filters increases accordingly, which can increase computational costs. |  |
|  |  | Model applying Fc-DenseNet that performs two tasks: crop and weed segmentation and stem detection using RGB and NIR images as input [5] | Two decoders share the output of one encoder to perform the two tasks, making it efficient. | As the two tasks are learned together, they may impede each other’s learning. |  |
|  |  | 4-stage model applying U-Net that performs crop segmentation and weed segmentation in stages [19]. | Segmentation is performed for only one class in one stage, thus improving segmentation accuracy. | As it is a sequential method, if learning in one stage is unstable, then learning in other stages is also impeded. |  |
|  |  | Model comprising a VGG-16-based encoder and U-Net-based decoder using R channel, NIR images, and NDVI images as input by concatenating them in the channel direction [20] | Concatenates images of various formats and uses them as input, thus improving segmentation accuracy. | An additional camera must be used to take NIR images, which reduces efficiency. |  |
|  |  | Modified U-Net model that uses crop and weed image patches as input [22]. | Extracts detailed features of the crop and weed shapes considering the local area using patches as input, and a data augmentation effect can be observed. | A patch may not have crops or weeds, and learning results may vary with the patch size. |  |
|  |  | Model comprising an encoder that connects UFAB and ResNet-50 and applies BAB for some blocks and a decoder that sequentially configures BAB, deconvolution, and SPRB [23]. | Improved segmentation accuracy by enabling the final output and the BAB output to be reflected in learning. | Each loss collides during backpropagation when using auxiliary loss, which can make learning unstable. |  |
|  |  | Two-stage model comprising two U-Net models, in which the 1st stage detects the object and the 2nd stage detects the crop, weed, and object [28]. | Reduced the difference between crop and weed segmentation accuracy while improving segmentation accuracy. | Performs crop, weed, and object segmentation in the 2nd stage based on the results of the object and background segmentation in the 1st stage; if the 1st stage is not properly learned, then neither is the 2nd stage. |  |
| Crop and weed segmentation  considering motion blur |  | Proposed method (WRA-Net ) | Improves crop and weed segmentation accuracy by restoring the images considering motion blur, making it suitable for application to real agriculture sites. | Sequentially performs blurred image restoration and crop and weed segmentation, resulting in a relatively long inference time. |  |
|  |  |  |  |  |  |

**Table S2.** Descriptions of three open datasets for training, validation, and testing.

| Dataset | Total | Train | Validation | Test |
| --- | --- | --- | --- | --- |
| CWFID | 60 | 45 | 5 | 10 |
| BoniRob | 492 | 400 | 30 | 62 |
| Rice seedling and weed | 224 | 160 | 20 | 44 |

**Table S3.** Hyperparameter setup used for training of WRA-Net

| Hyperparameters | Initial  learning rate | Minimum  learning rate | $\beta_{1}$ | $\beta_{2}$ | Batch size | Epochs |
| --- | --- | --- | --- | --- | --- | --- |
| Values | 10^-4^ | ${10}^{-7}$ | 0.9 | 0.999 | 2 | 450 |

**Table S4.** Hyperparameter setup used for training of U-Net

| Hyperparameters | Learning rate | $\beta_{1}$ | $\beta_{2}$ | Batch size | Epochs |
| --- | --- | --- | --- | --- | --- |
| Values | 10^-5^ | 0.9 | 0.999 | 2 | 150 |

**Table S5.** Comparison of segmentation accuracy for blurred data and restored data of various semantic segmentation models (BG means background)

| Before  Restoration | Model | $mIOU$ | Crop$IOU$ | Weed $IOU$ | BG $IOU$ | $Recall$ | $Precision$ | $F1 score$ |
| --- | --- | --- | --- | --- | --- | --- | --- | --- |
|  | U-Net  [7] | 0.6786 | 0.4647 | 0.5984 | 0.9726 | 0.7874 | 0.7985 | 0.7920 |
|  | DeepLabv3+  [53] | 0.6727 | 0.4157 | 0.6315 | 0.9709 | 0.7708 | 0.8079 | 0.7883 |
|  | SegNet  [16] | 0.6717 | 0.4591 | 0.5952 | 0.9608 | 0.8476 | 0.7419 | 0.7904 |
|  | Modified U-Net  [22] | 0.6524 | 0.4456 | 0.5411 | 0.9705 | 0.8024 | 0.7663 | 0.7810 |
| After  Restoration | U-Net  [7] | **0.7741** | **0.6247** | **0.7143** | **0.9833** | **0.8596** | **0.8775** | **0.8677** |
|  | DeepLabv3+  [53] | 0.7350 | 0.5321 | 0.6880 | 0.9793 | 0.8292 | 0.8539 | 0.8393 |
|  | SegNet  [16] | 0.7044 | 0.5094 | 0.6309 | 0.9728 | 0.8707 | 0.7615 | 0.8119 |
|  | Modified U-Net  [22] | 0.7450 | 0.5711 | 0.6814 | 0.9825 | 0.8457 | 0.8303 | 0.8376 |

**Table S6.** Summary of cases of ablation studies for proposed modules. “# mDSCB groups” means the number of mDSCB groups in Lite WRARB; the number of mDSCB modules in each group is shown in ( ).

| Cases | $\boldsymbol{\alpha}$ | attention gate | Deformable ResBlock | # mDSCB groups |
| --- | --- | --- | --- | --- |
| Case 1 |  | ✓ | ✓ | 4 (1, 2, 3, 4) |
| Case 2 | ✓ |  | ✓ | 4 (1, 2, 3, 4) |
| Case 3 | ✓ | ✓ |  | 4 (1, 2, 3, 4) |
| Case 4 | ✓ | ✓ | ✓ | 2 (1, 2) |
| Case 5 | ✓ | ✓ | ✓ | 2 (3, 4) |
| Case 6 | ✓ | ✓ | ✓ | 3 (1, 2, 3) |
| Case 7 | ✓ | ✓ | ✓ | 3 (2, 3, 4) |
| Case 8 (proposed) | ✓ | ✓ | ✓ | 4 (1, 2, 3, 4) |

**Table S7.** Summary of schemes of ablation studies according to motion blur application and restoration. The data used for training and testing in each scheme are indicated; original, blurred, and restored refer to the original data, motion-blurred data, and data restored by WRA-Net, respectively.

| Schemes | Training | Testing |
| --- | --- | --- |
| Scheme 1 | original | original |
| Scheme 2 | original | blur |
| Scheme 3 | blur | blur |
| Scheme 4 | original | restored |
| Scheme 5 (proposed) | restored | restored |

**Table S8.** Comparison of semantic segmentation accuracies according to the application of motion blur and restoration.

| Methods | $mIOU$ | Crop$IOU$ | Weed $IOU$ | BG $IOU$ | $Recall$ | $Precision$ | $F1 score$ |
| --- | --- | --- | --- | --- | --- | --- | --- |
| Scheme 1 | 0.7605 | 0.8489 | 0.4475 | 0.9853 | 0.8255 | 0.8585 | 0.8407 |
| Scheme 2 | 0.4321 | 0.3456 | 0.0468 | 0.9040 | 0.6707 | 0.4771 | 0.5556 |
| Scheme 3 | 0.7083 | 0.7978 | 0.3471 | 0.9802 | 0.7981 | 0.8169 | 0.7970 |
| Scheme 4 | 0.7228 | 0.8043 | 0.3817 | 0.9825 | 0.8013 | 0.8276 | 0.8134 |
| Scheme 5 | 0.7444 | 0.8269 | 0.4224 | 0.9838 | 0.8165 | 0.8420 | 0.8282 |

**Table S9.** Comparison of semantic segmentation accuracies according to motion blur application and restoration.

| Methods | $mIOU$ | Crop$IOU$ | Weed $IOU$ | BG $IOU$ | $Recall$ | $Precision$ | $F1 score$ |
| --- | --- | --- | --- | --- | --- | --- | --- |
| Scheme 1 | 0.7226 | 0.6311 | 0.5998 | 0.9368 | 0.8455 | 0.8235 | 0.8325 |
| Scheme 2 | 0.6038 | 0.4647 | 0.4319 | 0.9148 | 0.7920 | 0.7087 | 0.7469 |
| Scheme 3 | 0.6911 | 0.5758 | 0.5722 | 0.9253 | 0.8210 | 0.7983 | 0.8078 |
| Scheme 4 | 0.7066 | 0.6087 | 0.5771 | 0.9340 | 0.8364 | 0.8105 | 0.8215 |
| Scheme 5 | 0.7149 | 0.6236 | 0.5873 | 0.9340 | 0.8407 | 0.8153 | 0.8260 |

Table S10. Inference time of proposed method on desktop and Jetson embedded system (unit: s)

| Environment | Method | Inference time (per single image) |
| --- | --- | --- |
| Desktop | WRA-Net | 0.1850 |
|  | WRA-Net + U-Net | 0.2468 |
| Jetson embedded system | WRA-Net | 1.5529 |
|  | WRA-Net + U-Net | 2.3430 |

Table S11. Comparisons of number of parameters, GPU memory, FLOPs, MACs of WRA-Net and the state-of-the-art methods

| Methods | # parameters | GPU memory requirement  (unit: MiB) | FLOPs  (unit: G) | MACs  (unit : T) |
| --- | --- | --- | --- | --- |
| DeblurGANv2  [54] | $60.93\times{10}^{6}$ | 2,758 | 606.24 | 0.40 |
| HINet  [31] | $88.67\times{10}^{6}$ | 14,510 | 3,585.03 | 3.58 |
| MIMO-UNet  [55] | $6.81\times{10}^{6}$ | 14,046 | 1,410.57 | 1.41 |
| MPRNet  [56] | $20.13\times{10}^{6}$ | 13,192 | 13,870.31 | 14.54 |
| NAFNet  [57] | $17.11\times{10}^{6}$ | 3,240 | 388.70 | 0.36 |
| WRA-Net  (proposed) | $3.30\times{10}^{6}$ | 3,700 | 1,700.85 | 1.98 |
